# Synthetic Atrophy for Longitudinal Cortical Surface Analyses

**DOI:** 10.3389/fnimg.2022.861687

**Published:** 2022-06-02

**Authors:** Kathleen E. Larson, Ipek Oguz

**Affiliations:** ^1^Department of Biomedical Engineering, Vanderbilt University, Nashville, TN, United States; ^2^Department of Computer Science, Vanderbilt University, Nashville, TN, United States

**Keywords:** synthetic atrophy, cortical thickness, cortical segmentation, accuracy validation, registration

## Abstract

In the fields of longitudinal cortical segmentation and surface-based cortical thickness (CT) measurement, difficulty in assessing accuracy remains a substantial limitation due to the inability of experimental validation against ground truth. Although methods have been developed to create synthetic datasets for these purposes, none provide a robust mechanism for measuring exact thickness changes with surface-based approaches. This work presents a registration-based technique for inducing synthetic cortical atrophy to create a longitudinal ground truth dataset specifically designed to address this gap in surface-based accuracy validation techniques. Across the entire brain, our method can induce up to between 0.8 and 2.5 mm of localized cortical atrophy in a given gyrus depending on the region's original thickness. By calculating the image deformation to induce this atrophy at 400% of the original resolution in each direction, we can induce a sub-voxel resolution amount of atrophy while minimizing partial volume effects. We also show that cortical segmentations of synthetically atrophied images exhibit similar segmentation error to those obtained from images of naturally atrophied brains. Importantly, our method relies exclusively on publicly available software and datasets.

## 1. Introduction

Cortical thickness (CT) is an important image-based marker for measuring patterns in both healthy aging and neurodegenerative pathologies. Methods to quantify CT are categorized as either volumetric, where thickness is measured directly from a structural brain MRI, or surface-based, where thickness is measured as the distance between surface reconstructions of the segmented gray matter (GM) and white matter (WM) layers. Surface-based methods, while less computationally efficient than their volumetric counterparts (Jones et al., 2000; Das et al., 2009; Lee et al., 2011; Tustison et al., 2013), generally yield more accurate results due to reduced errors from partial volume effects and lower susceptibility to noise and topological defects (Clarkson et al., 2011). Although advancements have been made in developing robust surface-based methods for cortical segmentation and quantifying CT (Dale et al., 1999; Fischl and Dale, 2000; Han et al., 2004; Oguz and Sonka, 2014a,b; Oguz et al., 2015), determining the accuracy of observed measurements still remains challenging due to the difficulty of obtaining ground truth for experimental validation. This becomes an even greater setback for validation of longitudinal studies where exact spatial correspondences across timepoints are desirable yet elusive.

Validation of CT measurements from MRI scans was initially achieved by comparing thickness values to those obtained *via* histology (Meyer et al., 1996; Fischl and Dale, 2000); this is problematic due to differences between *in vivo* vs. postmortem tissue, MR vs. histology imaging differences, and 2D vs. 3D measurements. More recently, both longitudinal and cross-sectional studies often achieve validation of CT measurements by comparing thicknesses observed by a new pipeline to those from previously existing algorithms (Oguz and Sonka, 2014a; Tustison et al., 2014, 2017). However, this makes it difficult to show whether the proposed method offers any improvement in accuracy over the current state of the art in reference to ground truth. Another approach for evaluating CT methods is through test-retest validation (Fischl and Dale, 2000; Hutton et al., 2008; Tustison et al., 2014), which is a measure of reproducibility rather than accuracy, or by assessing the correctness of surface topology rather than anatomical accuracy (Dale et al., 1999; Reuter et al., 2012). Finally, another method is to compare surface placement in cortical segmentation results to manual landmarks (Han et al., 2004; Oguz and Sonka, 2014b) placed along the boundaries of the cortical ribbon within the image, such as those of the publicly available Johns Hopkins University (JHU) cortical validation dataset (Shiee et al., 2014).^1^ Unfortunately, these landmark datasets are generally limited to cross-sectional analyses; even if landmarks were manually placed in a longitudinal dataset, there would not exist exact correspondence between landmarks across timepoints. A better approach is to employ a synthetic dataset with known, ground truth changes in thickness and surface location at each point on the cortical surfaces.

Several methods for inducing synthetic deformations in brain images have been developed to validate techniques designed to measure *volumetric* cortical changes (Freeborough and Fox, 1997; Davatzikos et al., 2001; Karaçali and Davatzikos, 2006; Khanal et al., 2017; Xia et al., 2019; Bernal et al., 2021). For example, Freeborough and Fox implemented an image magnification technique to validate their boundary shift integral method for detecting volumetric loss. Davatzikos et al. synthetically induced cortical atrophy by simulating biomechanical deformations in a localized region to validate a voxel-based morphometry approach to atrophy detection. One of the most popular methods for inducing cortical atrophy is that of Karaçali et al., where a topology preserving deformation is used to induce a predetermined amount of volumetric change at each voxel in the image. More recent approaches include Xia et al. and Bernal et al., which both employ deep learning to simulate atrophy in structural MRI, and Khanal et al., which uses a biophysical model to generate cortical changes that are then induced with deformable registration. While these methods are all capable of generating longitudinal data with synthetically induced changes in CT, none of them also provide a robust mechanism for measuring exact thickness changes for surface-based approaches. Additionally, these volumetric approaches aside from that proposed by Karaçali et al. are likely to cause topological defects in the cortex.

In this paper, we present a method based on mathematical morphology and deformable registration to create a longitudinal ground truth dataset specifically designed for accuracy validation of *surface-based* CT measurements. Moreover, we demonstrate how we quantify the ground truth thickness changes using a method unbiased by previously existing thickness measurement techniques. We also show the applicability of our method to assess the accuracy of cortical segmentation and surface-based reconstructions. Firstly, we introduce the datasets used to develop and validate our methods. Next, we provide a detailed description of each step of the proposed synthetic atrophy pipeline, and demonstrate how we use cortical surface reconstructions to quantify the ground truth changes in CT. We then describe the experiments used to determine the degree and localization of cortical atrophy induced in different regions across the brain, and the extent to which accurate cortical segmentations can be produced from our synthetically atrophied data. Finally, we present the results of these experiments and discuss their implications for our methods.

## 2. Materials

We employ two publicly available datasets to develop and validate the methods presented in this paper. The first, which we use to develop our synthetic atrophy pipeline and validate it in the context of CT measurement, is the NITRC Kirby test-retest dataset (*n* = 21) of healthy adult data (Landman et al., 2011). For each subject in this dataset, there exists two separate sessions of 15 different image sequences; the two sessions are acquired within several hours of each other. Since minimal anatomical difference is expected between these two sessions, our analysis uses only the first sessions of the MPRAGE T1w and FLAIR images as the initial timepoint in our experiments and induces synthetic atrophy on these to create a second timepoint. The T1w images in this dataset were acquired with a resolution of 1.0 × 1.0 × 1.2 mm^3^ over an FOV of 240 × 204 × 256 mm^3^, and the FLAIR with 1.1 × 1.1 × 1.1 mm^3^ over an FOV of 242 × 180 × 200 mm^3^.

For the application of our methods to measuring cortical segmentation accuracy, we use the JHU cortical validation dataset (*n* = 10) (Shiee et al., 2014). This dataset contains five healthy controls (HC) and five multiple sclerosis (MS) subjects, with an MPRAGE T1w, FLAIR, T2w, and proton density image for each. Again, we use only the T1w and FLAIR modalities, which have the same resolution and FOV as the Kirby dataset. In addition to these images, each subject is associated with sets of fiducial landmarks denoting the cortical GM and WM surface placement. Three clusters of ten landmarks are placed by two different experts (referred to in this paper as experts A and B) within seven different brain regions: the calcarine fissure, cingulate gyrus, central sulcus, parieto-occipital sulcus, superior frontal gyrus, superior temporal gyrus, and Sylvian fissure. Each cluster of 10 landmarks is constrained to a single axial slice, but the three clusters within a region each lie in a separate slice. These clusters are provided in pairs for both GM and WM surfaces, such that a pair of clusters denotes the GM or WM borders of a given region within the same axial slice. Each hemisphere contains 7 of these pairs, which totals to 28 sets (7 regions × 2 hemispheres × 2 surfaces) of 30 landmarks (3 clusters × 10 landmarks) per subject per expert. Example pairs of GM/WM landmark clusters can be observed in **Figures 3A,C**, which displays one of the three left superior temporal (LST) pairs, or **Figures 7A,C**, which displays both the LST cluster and another cluster in the right calcarine (RCALC) fissure.

All subjects from both the Kirby and JHU datasets are processed using the FreeSurfer program (Dale et al., 1999) (version 6) with the “-FLAIRpial” option to produce a cortical parcellation (aparc+aseg.mgz) defined by the Desikan-Killiany Atlas (Desikan et al., 2006). We use this parcellation to produce a WM mask, a cortical GM ribbon mask, and a full brain mask. This full brain mask includes voxels corresponding to GM, WM, subcortical structures, and the ventricles, but excludes the cerebellum and brain stem. We use the WM defined in this atlas for our WM mask rather than FreeSurfer's volumetric WM segmentation so that the boundaries between the WM and GM labels are consistent. We also create a skull-strip mask from a thresholded skull-stripped brain image (brainmask.mgz), which differs from the “full brain mask” because it also contains voxels corresponding to CSF in addition to brain tissue. These resulting masks are all required for several steps of our synthetic atrophy pipeline. Lastly, prior to running our pipeline, we resample all images to an isotropic resolution that is determined such that no data is lost along the highest resolution axis; in our experiments this corresponds to 1 × 1 × 1 mm^3^ resolution.

## 3. Methods

### 3.1. Synthetic Atrophy Induction

The overall goal of our synthetic atrophy pipeline is to apply a localized deformation to a T1w image that will push the outer boundary of a GM region in toward the WM to simulate localized atrophy. We achieve this by first creating a local atrophy “target” using a series of binary morphology operations on an ROI within the cortical ribbon. We then compute a restricted, deformable registration from the original ROI to the atrophied target to create a smooth deformation field. This allows us to obtain a transformation with point-to-point correspondences between timepoints along with the final atrophied image.

**Figure 1:**
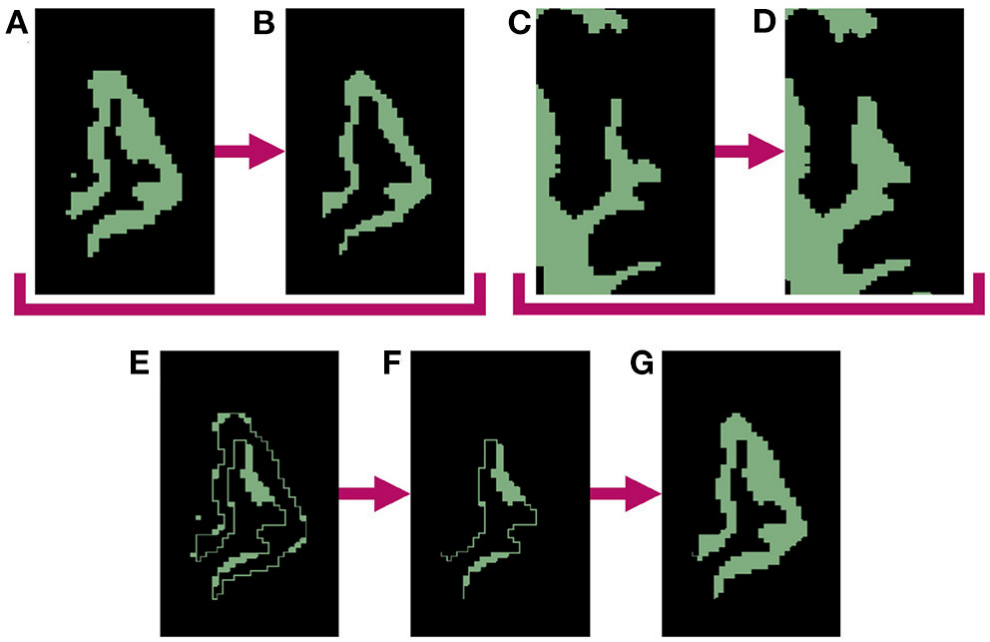
Binary mathematical morphology operations used to induce 1-voxel amount of atrophy in the left superior temporal gyrus (LSTG). **(A)** Mask of original LSTG. **(B)** Binary erosion of original LSTG mask **(A)** with a kernel radius of 1 voxel. Note this represent atrophy on both the inner and outer parts of the GM region, which will create undesirable holes between the WM and GM. **(C)** Mask of original WM. **(D)** Binary dilation of original WM mask **(C)** with a kernel radius of 1 voxel. **(E)** Original LSTG mask minus eroded LSTG mask **(A−B)**. **(F)** Dilated WM mask times the difference between original and eroded LSTG masks **(D × E)**. This represents the holes between the WM and GM created by the erosion operation. **(G)** Final mask of atrophied ROI, with the holes filled **(B+F)**.

The process begins by generating a cortical parcellation from the target image and selecting a specific gyrus within this label map to serve as the region of interest (ROI) for atrophy. Although in our experiments we use FreeSurfer (Dale et al., 1999) to produce the parcellation defined by the Desikan-Killiany Atlas (Desikan et al., 2006), our methods are generalizable to any parcellation. We create a binary mask of the selected ROI, which we upsample by 400% in each direction to avoid introducing partial volume artifacts. We then perform a set of binary mathematical morphology operations on the high-resolution mask using the publicly available Insight Toolkit (ITK)^2^ library. This series of operations simulates localized atrophy by first removing a 1-voxel border around the entire ROI and then reinserting voxels adjacent to WM, thus effectively “sloughing off” the outermost layer of voxels along only the GM/CSF interface without altering the GM/WM interface. Figure 1 displays this entire series of binary operations, which can be repeated as many times as needed to produce the desired amount of total atrophy. Specifically, for each iteration, we perform the following steps:

1. Apply a binary erosion filter with a kernel of radius 1 × 1 × 1 voxels to the GM ROI (Figure 1A→Figure 1B).
2. Apply a binary dilation filter with the same kernel to the WM mask. This preserves the GM/WM interface (Figure 1C→Figure 1D).
3. Subtract the eroded GM mask from the original to obtain a mask of the removed voxels (Figure 1A−Figure 1B=Figure 1E).
4. Select from the mask obtained in step 3 only voxels within the dilated WM mask to extract all voxels lying on the GM/WM border (Figure 1D × Figure 1E=Figure 1F).
5. Add back the border voxels extracted in step 3 to the eroded mask from step 1 (Figure 1B+Figure 1F=Figure 1G).

Once complete, these binary morphology operations yield a GM ROI with an unchanged WM boundary and a outer border eroded by one voxel, which in practice, also expands the CSF by one voxel. Each subsequent iteration erodes another single layer of GM voxels; this can proceed until the entire GM ribbon is removed from the ROI. Note that because we must dilate the WM to meet the eroded GM in order to preserve the boundary between the two tissues (step 2), this limits each iteration to eroding up to one half of the thickness of the original ROI. This limitation would persist even with a larger erosion kernel, and the morphology operations would fail if we used a kernel sized larger than half the tissue thickness. However, by only eroding one voxel per iteration, we bypass this issue and can atrophy deeper into the ROI. We refer to the number of iterations as the “effective erosion kernel size” because *k* number of iterations removes an equal number of voxels to a single erosion operation that uses a kernel of size *k* × *k* × *k* voxels. The end product of this series of iterations is the local atrophy target.

**Figure 2:**
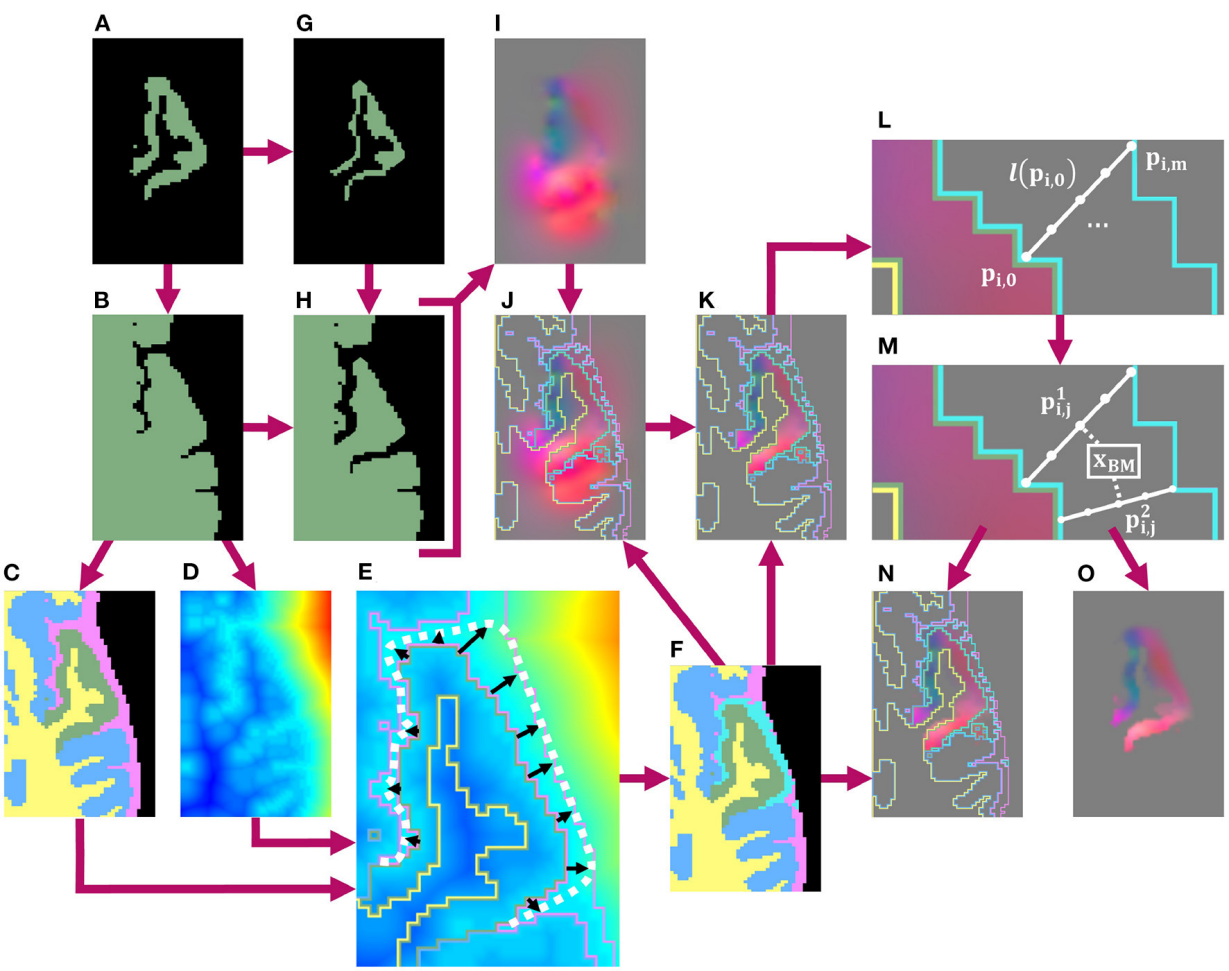
Schematic of pipeline to produce the masked and blurred deformation field for synthetic atrophy in the left superior temporal gyrus (LSTG) with 4 iterations of the binary mathematical morphology operations shown in Figure 1. **(A)** Original LSTG. **(B)** Original LSTG **(A)** with surrounding GM/WM, also referred to as the full brain mask of the original timepoint. **(C)** Full brain mask of original timepoint parcellated with 4 labels: the original ROI (green), WM (yellow), surrounding GM tissue (blue), and CSF (pink). **(D)** Signed distance transform (SDT) of original full brain mask. **(E)** Example of traveling along lines (black) normal to the GM/CSF boundary that extend to either the outer boundary of the CSF (pink), or the medial lines between neighboring gyri defined by the monotonic increase of the SDT **(D)** The full approximated boundary is denoted by the dotted white line. **(F)** Label map shown in **(C)** with added blur region (teal). **(G)** Atrophied LSTG (local atrophy target). **(H)** Atrophied LSTG (B) with surrounding GM/WM. **(I)** Deformation field obtained by registering the original LSTG + surrounding tissue mask **(B)** to the atrophied LSTG + surrounding tissue mask **(H)**. **(J)** Deformation field **(I)** with label mask **(F)** as overlay. **(K)** Deformation field masked to the original ROI **(A)**, with label mask **(F)** as overlay. **(L)** Example of defining *m* number of equally spaced points along a line *l*(*p*_*i*,0_) oriented normal to the GM/CSF boundary and extending from *p*_*i*,0_ to *p*_*i,m*_. **(M)** Example of interpolating the field value at a voxel *x*_*BM*_ within the blur mask using the *n* closest points to the center of that voxel. These points are constrained to lie on unique lines. In this 2D example, only 2 lines are used, but in 3D, the value is interpolated using the 4 closest lines. **(N)** Final deformation field after masking and outward smoothing, overlaid with label map from **(F)**. The field inside the blur mask (teal) is a smooth transition from the original, unchanged field inside the ROI to the edge of the CSF or medial boundaries between gyri. **(O)** Final deformation field after masking and outward smoothing with no overlay.

Once we thus obtain a binary mask for the local atrophy target, we next calculate a transformation to deform the T1w image accordingly, as well as any additional associated data such as T2w or FLAIR images. To achieve this, we first create high-resolution, full brain masks for both the original and atrophied timepoints. The original brain mask is obtained by thresholding a 400% upsampled cortical parcellation from which the ROI was selected (Figures 2A–C), and the atrophied brain mask is obtained by substituting the eroded ROI mask for the original in the thresholded parcellation (Figures 2G,H). We deformably register the original, full brain mask to the atrophied using the publicly available Greedy software (Yushkevich et al., 2016), resulting in a deformation field from the original to atrophied timepoint (Figure 2I). This registration is performed at four scales (100/100/50/100 iterations per scale, respectively) and using the mean squared difference as the similarity metric.

Next, we mask the resulting deformation with the original ROI and blur it outwards into the CSF within a custom blur mask. Creating this mask requires two inputs: a preliminary label map (Figure 2C) and the signed distance transform (SDT) (Figure 2D) of the full brain mask (Figure 2B). This preliminary label map parcellates the full brain mask into voxels inside the ROI (green), the rest of the GM ribbon (blue), the WM (yellow) as defined in the original DK atlas, and CSF (pink) defined using the skull-strip mask. Using these two images (Figures 2C,D), we define the blur mask (teal region in Figure 2F) as the region bound by the GM/CSF border of the ROI, the outer edge of the CSF, and the medial lines between neighboring gyri calculated using the monotonic increase of the SDT (Figure 2E).

Inside this blur mask, we assign values to voxels such that we create a smooth transition from the deformation field values inside the GM ROI to zero-valued voxels at the edge of the skull-strip mask or medial lines (Figures 2L,M). Let $\vec{f}\left({\text{p}}_{i,0}\right)$ be the value of the deformation field at a point **p_i,0_** that lies on the interface between the ROI and the blur mask. Let *l*(**p_i,0_**) be the line oriented normal to the GM/CSF interface at **p_i,0_** and that extends from **p_i,0_** to another point **p_i,m_** on the opposite edge of the blur mask (i.e., either at the edge of the skull-strip mask or on a medial line). Along *l*(**p_i,0_**), we sample equally spaced points **p_i,1_**, …, **p_*i,m*−1_**. Each of the *m* + 1 points on *l*(**p_i,0_**) is then associated with a vector $\vec{f^{\prime }}\left({\text{p}}_{i,j}\right)$ such that


(1) $$\begin{array}{l} \vec{f^{\prime }}\left({\text{p}}_{\text{i},\text{j}}\right)=\frac{m-j}{m}\vec{f}\left({\text{p}}_{\text{i},0}\right)\;\left(0\leq j\leq m\right) \end{array}$$

This yields a series of vectors that decrease linearly from $\vec{f^{\prime }}\left({\text{p}}_{i,0}\right)=\vec{f}\left({\text{p}}_{i,0}\right)$ to $\vec{f^{\prime }}\left({\text{p}}_{i,m}\right)=\vec{0}$, which we calculate for each line across the entire interface between the ROI and the blur mask (Figure 2L). Using vectors from these series, we can interpolate field values $\vec{f}\left({\text{x}}_{BM}\right)$ at each voxel **x_BM_** inside the blur mask (Figure 2M). If we define ${\text{p}}_{i,j}^{1}\ldots {\text{p}}_{i,j}^{n}$ as the *n* closest points to the center of **x_BM_**, and $\vec{f^{\prime }}\left({\text{p}}_{i,j}^{1}\right)\ldots \vec{f^{\prime }}\left({\text{p}}_{i,j}^{n}\right)$ as their corresponding vectors, then we can express the interpolated value $\vec{f}\left({\text{x}}_{BM}\right)$ as


(2) $$\begin{array}{l} \vec{f}\left({\text{x}}_{\text{BM}}\right)=\frac{1}{n}\overset{n}{\underset{k=1}{\sum }}d\left({\text{x}}_{\text{BM}},{\text{p}}_{\text{i},\text{j}}^{\text{k}}\right)\cdot \vec{f^{\prime }}\left({\text{p}}_{\text{i},\text{j}}^{\text{k}}\right) \end{array}$$

Here, $d\left({\text{x}}_{BM},{\text{p}}_{i,j}^{k}\right)$ denotes the distance between the center of **x_BM_** and the point ${\text{p}}_{i,j}^{k}$. We require that each ${\text{p}}_{i,j}^{k}$ lie on a unique *l*(**p_i,0_**); even if the center of **x_BM_** is closest to multiple points on the same line, $\vec{f}\left({\text{x}}_{BM}\right)$ will still be calculated using one point from *n* distinct *l*(**p_i,0_**). In our experiments, we use *n* = 4. This entire process of obtaining the deformation field by registering the original full brain mask to its atrophied counterpart, followed by masking and smoothing the field with a custom blur mask, is detailed in Figure 2.

Masking and smoothing the field in this way offers several advantages over other techniques, such as a simple Gaussian blur. Firstly, we create a smooth transition between the deformed and original image regions; this ensures that the boundary of the GM is still deformed even if the edge of the ROI does not quite extend to the edge of the GM in the input T1w image (e.g., due to inaccuracies in input FreeSurfer parcellation). Constraining the blur prevents the transformation from extending into and deforming neighboring regions, which would result in the GM expanding within those areas. Finally, leaving the deformation field inside the ROI unchanged ensures that the deformation performs as expected.

After calculating the final deformation field with this custom blur operation, we apply it to the original T1w image to artificially induce localized cortical atrophy. This yields a set of two timepoints with known changes at each location in the images. Any other images, such as FLAIR or T2w, can be co-registered to the T1w image and undergo the same deformation to induce the same synthetic atrophy in these modalities.

### 3.2. Cortical Surface Thickness Change

After creating our set of images, where one timepoint is the original data and the other synthetically atrophied, our next step is to quantify the true change induced in CT. By construction, our method induces surface erosion perpendicular to the surface, whereas the direction of thickness measurement might be at a slight angle based on the local cortical geometry. Because of this discrepancy, we expect that the true change in thickness will be less than the effective size of the erosion kernel. Thus, to determine the localized, ground truth changes in CT, we create surface representations for each timepoint and measure the corresponding difference in thickness in the deformed region. We obtain these surfaces by performing the 3D Slicer (Fedorov et al., 2012) implementation of the marching cubes algorithm^3^ (Lorensen and Cline, 1987) on the WM and whole brain GM masks (rather than just the ROI) of the original image. We use a smoothing factor of 10 and 0% decimation for input parameters, and then remove any topological defects in the surface such as holes or handles (Jaume et al., 2005). This resulting surfaces are warped with the same deformation field used to transform the image to yield corresponding surfaces for the atrophied timepoint. Although the marching cubes algorithm does not necessarily produce a topologically accurate mesh representation, we employ this technique rather than a specific cortical surface reconstruction pipeline (such as FreeSurfer) so that our results are not biased toward any specific reconstruction method.

Finally, we define the ground truth change in CT as the average between the difference between the distances between the original and atrophied surfaces. For each vertex on each original surface (either GM or WM), we calculate the shortest, signed distance from that vertex to a point on the corresponding atrophied surface. Note that this point can lie anywhere on the atrophied surface and is not necessarily coincident with one of its vertices. Next, we average these distances across the entire ROI to yield a mean surface displacement value, and take the difference between the mean GM displacement and the mean WM surface. We then repeat these calculations starting at each point on the atrophied surfaces and finding the shortest, signed distances to their original counterparts. The true change in thickness is thus defined as the average between the difference in surface displacements traveling from the original to atrophied surfaces, and the different in displacements traveling from the atrophied to the original. We call this definition the Mean Surface Displacement Difference (MSDD). In this analysis, all mesh warping, topological corrections, cortical surface parcellations, and thickness measurements are obtained using the Visualization Toolkit (VTK)^4^ libraries.

### 3.3. Longitudinal Cortical Segmentation Accuracy

Recall that we develop our methods with two intended applications: validation of surface-based CT measurement *and* of cortical segmentation. Thus, after establishing a method for measuring the exact induced thickness change, we want to demonstrate how data from our methods can be employed to quantify segmentation accuracy as well in the presence of cortical atrophy. We also want to determine whether cortical segmentation pipelines can segment our synthetic images well or whether they encounter issues such as local blurring of boundaries due to interpolation. We accomplish this by inducing synthetic atrophy in the JHU dataset; we deform both the images and the landmarks themselves to match the new cortical boundaries. We use the original and deformed landmarks sets to measure the accuracy of FreeSurfer cortical surface reconstructions of the respective timepoints.

**Figure 3:**
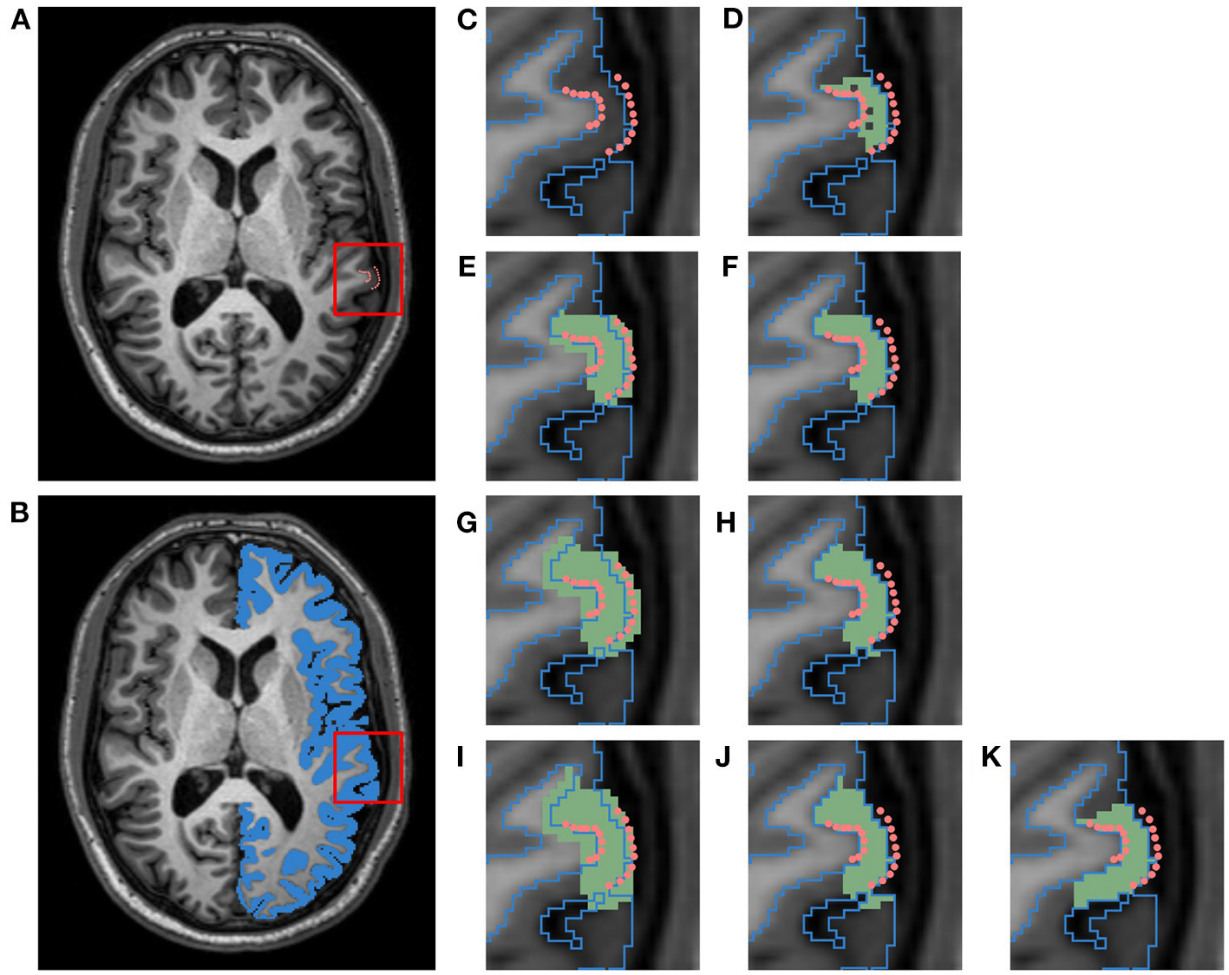
Schematic of pipeline to produce a label map specific to the left superior temporal (LST) fiducial landmarks. This data belongs to a subject from the HC cohort of the JHU cortical validation dataset. **(A)** T1w image with manual fiducial landmarks (pink). **(B)** T1w image overlayed with a mask of the left cortical GM ribbon (blue). **(C)** Close up of landmarks in T1w image overlayed with GM ribbon mask outline. **(D)** Initial ROI (green) for image and landmark deformation, obtained by filling in voxels between corresponding pairs of WM and GM landmarks. **(E)** ROI dilated with 1 × 1 × 1 voxel kernel (first iteration). **(F)** ROI masked with cortical GM ribbon (first iteration). **(G)** Dilated ROI (second iteration). **(H)** Masked ROI (second iteration). **(I)** Dilated ROI (third iteration). **(J)** Masked ROI (third iteration). **(K)** Final ROI after manual cleanup of adjacent gyri.

Because we are only interested in measuring cortical accuracy at the fiducial landmarks, we create a unique cortical parcellation for each subject based on the locations of its landmark clusters. This label map contains ROIs that are each centered around a single cluster of GM/WM landmarks (recall there are 3 such clusters per region in the JHU dataset, where each cluster consists of 10 landmarks per surface and is constrained to a single axial slice). Figure 3 illustrates the process of creating a single ROI for one cluster of the left superior temporal (LST) region (Figure 3C). The ROI is defined by first filling in the voxels within the image that lie between pairs of corresponding GM and WM landmarks (Figure 3D). Because each cluster of landmarks lies within a single axial slice, this step yields a 1-voxel-thick mask with no extra voxels surrounding the landmarks. Next, we want to pad this initial ROI so that the deformation moving the landmarks is not affected by boundary effects. To accomplish this, we iterate between (1) dilating this initial mask outward with a 1 × 1 × 1 voxel kernel and (2) applying a cortical ribbon mask to constrain the ROI to within the GM (Figures 3E–J). In other words, we use voxels between corresponding GM and WM landmarks as a starting seed, and flood the ROI outwards within the cortical ribbon. This adds a buffer region on either side of the landmark cluster, and of 3 axial slices on both sides of the initial slice; this also removes any holes existing within the original ROI. We note that this iterative dilation and masking procedure can result in the ROI expanding into adjacent tissue. To alleviate this, each ROI is manually edited using 3D Slicer^5^ to remove any voxels residing in adjacent gyri, and to ensure smoothness between axial slices (Figures 3J,K). Using this procedure, we generate a total of 42 ROIs (21 per hemisphere), as each of the seven regions contain landmark clusters in three different axial slices.

After manual editing, we induce synthetic atrophy in each ROI with 2 iterations of the binary morphology operations shown in Figure 1. We use 2 iterations to induce consistent amounts of change in each region, and to make sure that at no location in any ROI the GM is fully eroded, as this would not occur during actual neurodegeneration. With the eroded atrophy target, we perform the registration step using the Greedy software with the same parameters as before (four levels, 100/100/50/100 iterations per level, and mean squared difference as the similarity metric). We calculate these individual deformation fields for all 42 ROIs, and combine them into a single deformation field. Let *T*_*j*_(1 ≤ *j* ≤ 42) denote these individual transformations, and let *C* denote the composite field. Let ${\vec{v}}_{x,j}$ be the value of the deformation field *T*_*j*_ at voxel *x*. There are three possible scenarios for the corresponding ${\vec{v}}_{x,C}$ in *C*:

1. If ${\vec{v}}_{x,j}=\vec{0},\forall j$, then ${\vec{v}}_{x,C}=\vec{0}$.
2. If there exists a unique *i* such that ${\vec{v}}_{x,i}\neq \vec{0}$ and $\forall_{j}[\left(j\neq i\right)\to \left({\vec{v}}_{x,j}=\vec{0}\right)$], which happens when *x* is within a single non-overlapping ROI, then ${\vec{v}}_{x,C}={\vec{v}}_{x,i}$.
3. If there exist multiple non-zero valued ${\vec{v}}_{x,j}$, which can happen because the blurred regions are not necessarily non-overlapping, then, ${\vec{v}}_{x,C}$ is calculated as the average of all non-zero valued ${\vec{v}}_{x,j}$ at voxel *x*.

These operations are all computed using ITK.

Finally, the composite field *C* is applied to the T1w and FLAIR images and to the associated fiducial landmarks. Here, the images are deformed using the Greedy software and the landmarks with VTK. This creates a set of images accompanied by cortical landmarks with exact correspondence between timepoints, which can be used to evaluate the accuracy of cortical surface reconstruction methods in a longitudinal setting.

### 3.4. Validation Experiments

#### 3.4.1. Localization of Cortical Atrophy

The goal of our first experiment is to determine if the transformation is indeed constrained as desired, or if it bleeds into the surrounding voxels and deforms the GM outside the ROI. To investigate this, after calculating thickness at each vertex, we compute a mean thickness value for 3 different cortical regions: inside the atrophied ROI, within a 4 × 4 × 4 voxel neighborhood surrounding the ROI, and everywhere else on the surface. Any significant change in thickness detected within the neighborhood surrounding the ROI would indicate that our transformation is not constrained to the target ROI as intended. We separate this surrounding neighborhood because otherwise any measured changes would be smoothed away if averaged together with the entire brain outside the target ROI. **Figure 6C** displays an example of this label map projected onto a cortical surface. Finally, changes in thickness were computed as the difference in mean thickness within each of the three regions. By taking the difference of the average thickness within each region instead of the average of the difference across pairs of vertices, we allow for the application of this method to CT pipelines that may not guarantee vertex-vise correspondence between timepoints.

#### 3.4.2. Extent of Induced Localized Cortical Atrophy

Although we can fully erode the GM ROI using the series of binary morphology operations described in Figure 1, the regularization terms used in the subsequent deformable registration step will likely not allow for the total collapse of the GM ribbon. Thus, our next experiment aims to determine the extent to which we can induce localized atrophy in ROIs throughout the entire cortex in practice. We tested our pipeline in each of the 62 cortical regions (33 per hemisphere) in the Desikan-Killiany (DK) atlas by varying the amount of target (intended) atrophy induced in each ROI from 1 to 12 voxels in the upsampled mask images. Because these upsampled masks have a resolution of 0.3 × 0.25 × 0.25mm^3^, each iteration induces approximately $\sqrt{0.3^{2}+0.25^{2}+0.25^{2}}=0.46\text{mm}$ of thickness change, resulting in a total change of about 5.5mm over 12 iterations. Note that this is intended to result in complete atrophy within the cortical region (which would not be expected to occur in a realistic dataset, except possibly in surgical removal scenarios) to test the limits of the atrophy pipeline. For each iteration of atrophy, we measure the mean change in CT using our MSDD definition of thickness change within each cortical ROI; this allows us to assess how the CT changes in individual ROIs with each iteration and the maximum extent to which we can synthetically induce localized atrophy. We also measure these changes with the symmetric closest point distance (SCPD), which defines CT at each vertex as the average of the distances between (1) the initial GM vertex and the closest point on the WM surface, and (2) that point on the WM surface and its closest point on the GM surface. Note that similar to the MSDD other than the initial vertex, these points are not constrained to vertices and can fall anywhere on the GM or WM surfaces. By comparing changes in CT measured with the MSDD and SCPD definitions, we can determine how our findings match those obtained with an established and widely used technique.

#### 3.4.3. Effect on Longitudinal Cortical Segmentation Accuracy

In this experiment, we aim to determine the usability of our methods in the context of validating the accuracy of the cortical segmentation methods. We explore this by inducing synthetic atrophy in the JHU cortical validation dataset using the custom cortical parcellations generated from the landmarks associated with each subject (Section 3.3). We use both the cross-sectional and longitudinal workflows of the FreeSurfer program to generate cortical surface representations of the original and atrophied timepoints, and measure the segmentation error of the resulting surfaces with respect to landmark placement. In the cross-sectional pipeline (Dale et al., 1999), each timepoint is segmented independently; in the longitudinal pipeline (Reuter et al., 2012), FreeSurfer first combines all timepoints to create a subject-specific template and then separately segments each timepoint using this template for initialization. We hypothesize that if our synthetic atrophy methods are significantly corrupting the data, e.g., with blurring artifacts, this would result in more error in the jointly initialized longitudinal pipeline compared to the cross-sectional pipeline.

After creating the synthetic dataset and surface reconstructions, we calculate the unsigned and signed segmentation errors for each landmark cluster. These errors are obtained by measuring the average distance from each of the 30 landmarks on each surface within the cluster to the corresponding cortical surface. We determine the sign of the distance by generating high resolution, binary masks for each surface: the distance is positive if the landmark resides inside the mask, and negative if outside. We expect that FreeSurfer will perform worse in atrophied data than in healthy data, regardless of whether the atrophy is synthetic or naturally occurring. To test this, we compare segmentation error between the healthy and MS cohorts within the dataset; the MS subjects present varying degrees of (natural) cortical atrophy. If there is no significant difference in FreeSurfer error between synthetically atrophied healthy data and the original MS data, then we can conclude that our method does not induce significant artifacts or error in the images or landmark placements. We use a one-way ANOVA (α = 0.05) to test for significant differences between errors for the original and synthetic timepoints for the HC and MS data (4 cohorts per test). These tests are conducted separately for each region (7 regions × 2 hemispheres × 2 surfaces, for a total of 28), expert (A or B), processing type (cross-sectional or longitudinal), and error type (unsigned or signed). We conduct a similar set of ANOVA tests for these data to identify significant differences between the two processing types, and between the two sets of experts (4 sets total). These tests are conducted separately for each region (28 total), data type (original of synthetic), subject type (HC or MS), and error type (unsigned or signed).

## 4. Results

### 4.1. Qualitative Evaluation of Atrophied Images

**Figure 4:**
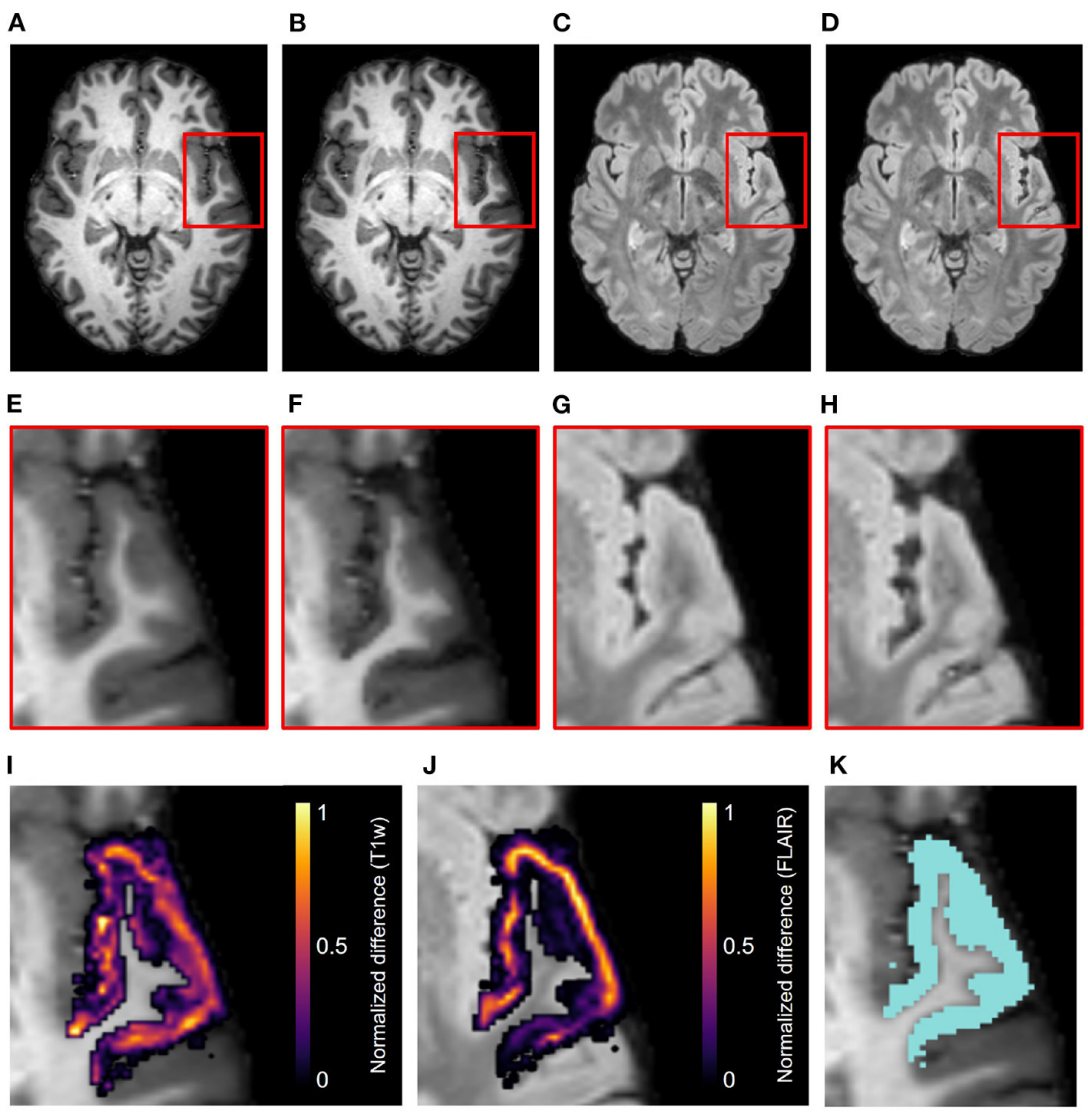
Example results of the synthetic atrophy pipeline using the NITRC Kirby dataset and four iterations of binary morphology operations to induce atrophy. **(A)** Skull-stripped original T1w image. **(B)** Skull-stripped T1w image with synthetically induced atrophy. **(C)** Skull-stripped original FLAIR image. **(D)** Skull-stripped FLAIR image with synthetically induced atrophy. **(E–H)** Close-up of ROI (LSTG) in the images of the first row. **(I)** Difference image between the original and atrophied T1w timepoints **(E,F)** overlayed on the original T1w image. **(J)** Difference image between the original and atrophied FLAIR timepoints **(G,H)** overlayed on the original FLAIR image. For both difference images, all zero-valued voxels are rendered transparent. **(K)** Close up of ROI (blue) overlayed onto original T1w image.

Figure 4 displays the axial view of an example set of T1w and FLAIR images. The original images are from the NITRC Kirby dataset (Figures 4A,C, and atrophy was synthetically induced (Figures 4B,D) in the left superior temporal gyrus (LSTG) (Figure 4K). In this example, we used an effective erosion kernel of *k* = 4 (4 × 4 × 4 voxels), corresponding to 4 iterations of binary morphology operations, to compute the synthetic atrophy deformation field. We observe a noticeable decrease in CT between the two timepoints (see Figures 4E–H for close-ups), with no significant changes in the GM surrounding LSTG, or at the GM/WM interface. This visually apparent localization is further supported by the difference image between the original and atrophied timepoints for each modality (Figures 4I,J). In these images, any zero-valued voxel is displayed as transparent. Thus, we see that the deformation is only affecting the ROI and the mask used during the blurring process (Figure 2F). We also see that the atrophied FLAIR image corresponds well to its T1w counterpart, indicating that we can apply the deformation obtained from a T1w image to atrophy additional modalities associated with the subject, as expected.

### 4.2. Localization and Extent of Cortical Atrophy

**Figure 5:**
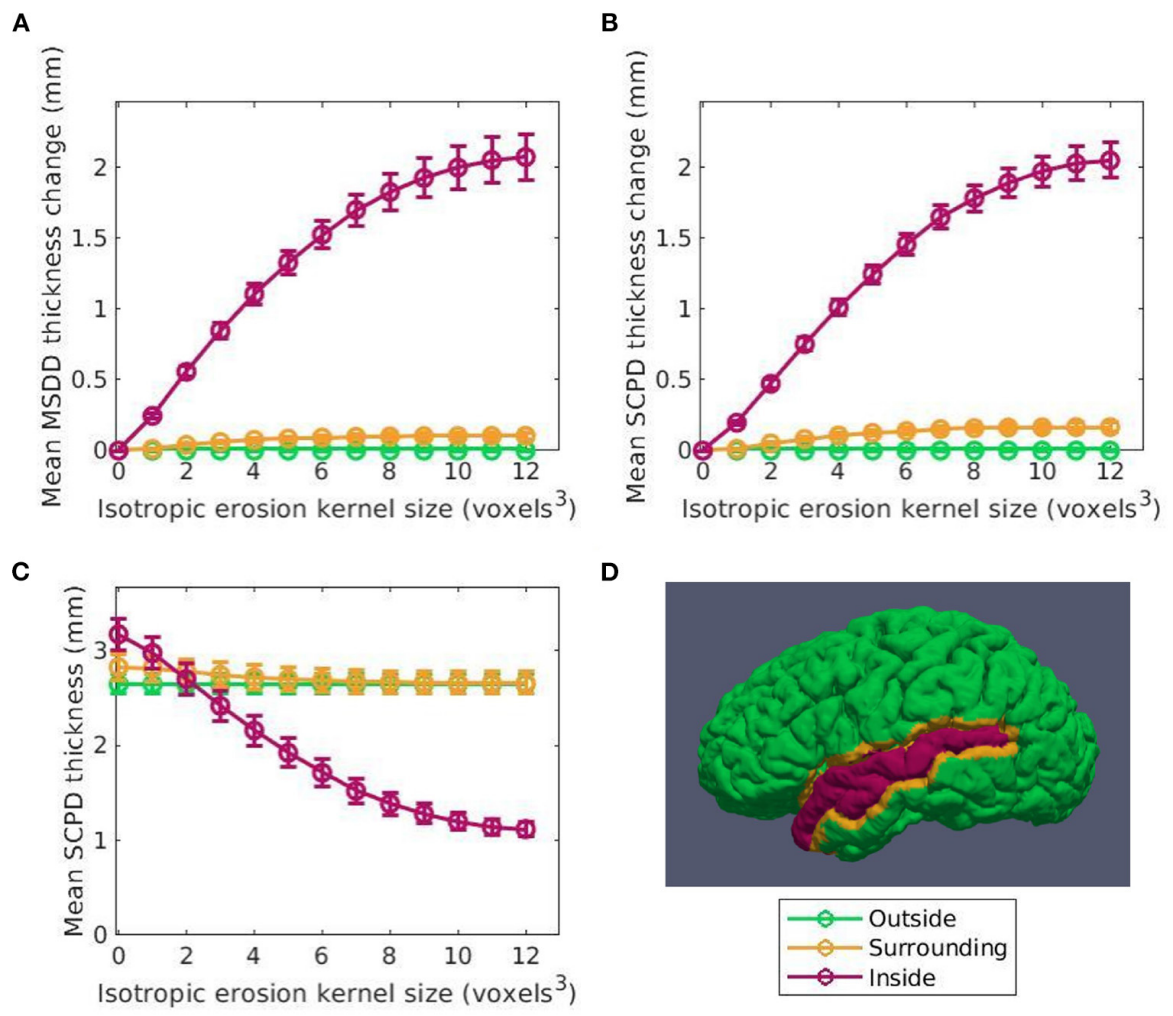
Thickness change averaged across subjects between the original and synthetic atrophied timepoints as a function of isotropic erosion kernel size in the 3 regions studied for the left superior temporal gyrus (LSTG) ROI. Data shown in pink corresponds to voxels inside the ROI is shown in pink, in yellow to the 4 × 4 × 4 voxel dilation neighborhood, and in green to the rest of the cortex. **(A)** Mean thickness change measured using the mean surface displacement difference (MSDD). **(B)** Mean thickness measured using the symmetric closest point distance (SCPD). **(C)** Mean change in thickness measured with SCPD. These values closely match the MSDD changes measured with shown in panel A. **(D)** Example label map of a single subject in the Kirby dataset showing the vertices inside the ROI, surrounding the ROI, and outside the ROI. These labels are projected onto a cortical surface generated using the marching cubes algorithm on the full brain mask corresponding to that subject.

Figure 5 shows the relationship between the induced thickness changes within the LSTG of our marching cubes generated surfaces and the number of atrophy iterations. The colors of the data in Figures 5A–C correspond to the labels overlaid as a cortical parcellation onto an example marching cubes surface in Figure 5D: inside the LSTG (pink), within the surrounding 4 × 4 × 4 voxel dilation neighborhood (yellow), and across the rest of the cortex (green). Figures 5A,B show the average thickness change across all subjects measured using the MSDD and SCPD formulas, respectively, while Figure 5C shows the actual SCPD thickness. The MSDD yields a higher change in thickness inside the ROI (pink) than the SCPD, but a lower change in the surrounding region (yellow). This is likely because the SCPD maps a vertex on the GM surface to a vertex on the WM, and then maps that vertex to a second GM vertex. If the second GM vertex corresponds to the surrounding region while first vertex corresponds to the desired ROI, then reported thickness change will be less than if all three vertices were constrained to the ROI. Likewise, if the second corresponds to the ROI while the first corresponds to the surrounding area, the reported change will be higher. This discrepancy between measurements inside the ROI (pink) also agrees with a finding in a previous CT study that the SCPD may underestimate thickness compared to alternate surface-based methods (Oguz and Sonka, 2014a). The MSDD and SCPD both found that thickness within the surrounding (yellow) region remains relatively stable as erosion kernel size increases, the MSDD more so than the SCP; this indicates that the atrophy induced by our methods is highly localized and constrained to the desired region. These thickness changes seem unaffected by the underestimation tendencies of the SCPD, most likely because they are much less pronounced than inside the ROI (pink). Finally, as expected, both methods report no change in thickness across the rest of the cortex (green). These curves shown in Figure 5 serve as a representative example for the atrophy trends across the entire cortex.

**Figure 6:**
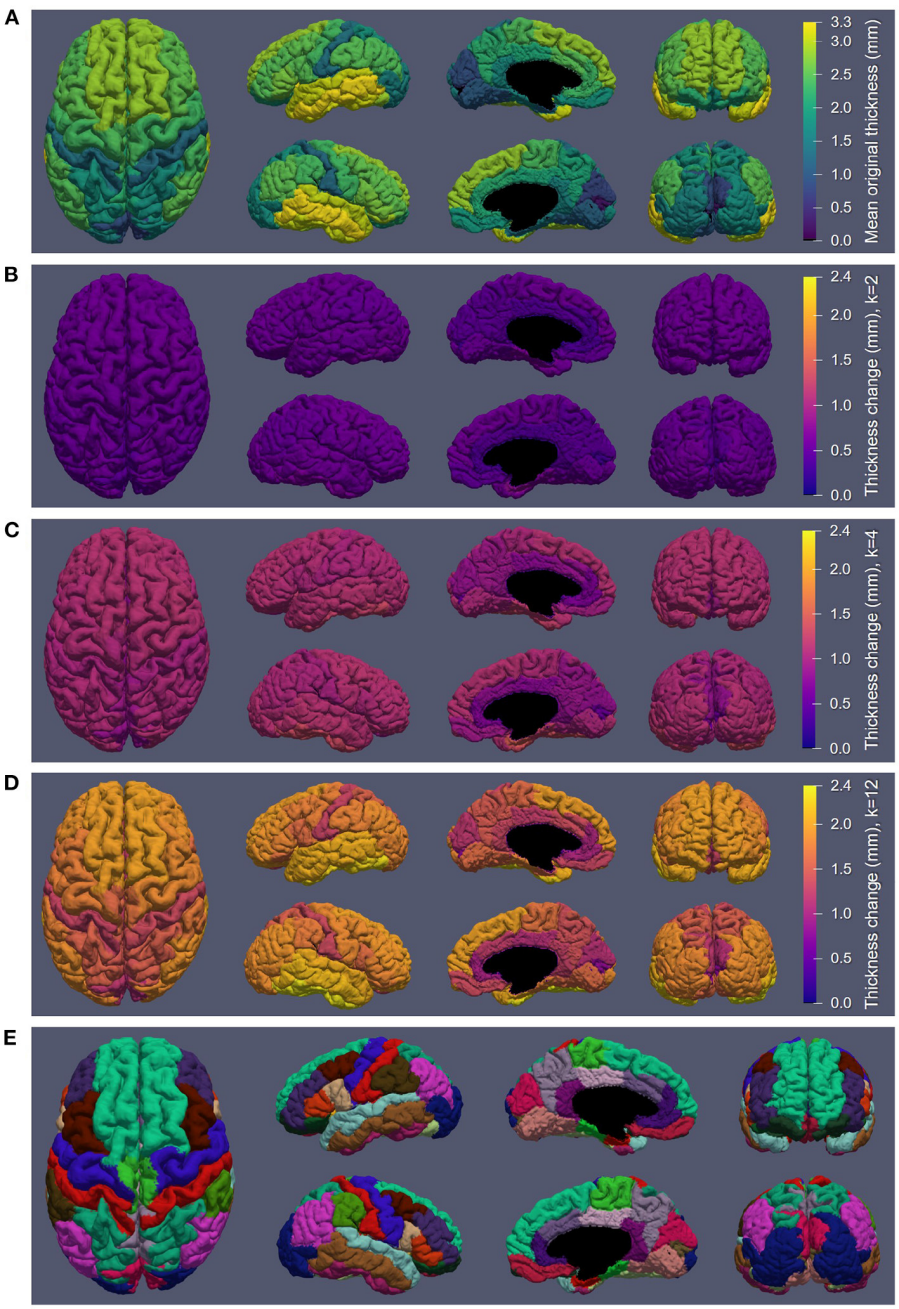
Synthetic atrophy results averaged across subjects mapped onto an example cortical GM surface obtained from the original timepoint using the marching cubes algorithm. **(A)** Mean original thickness for each region measuring using the symmetric closest point distance (SCPD) formula. **(B)** Thickness change measured with using the mean surface displacement distances (MSDD) after *k* = 2 iterations of erosion. **(C)** MSDD thickness change after *k* = 4 iterations of erosion. **(D)** MSDD thickness change after *k* = 12 iterations of erosion, showing the maximum amount we can atrophy in each region. **(E)** DK atlas (Desikan et al., 2006).

**Figure 7:**
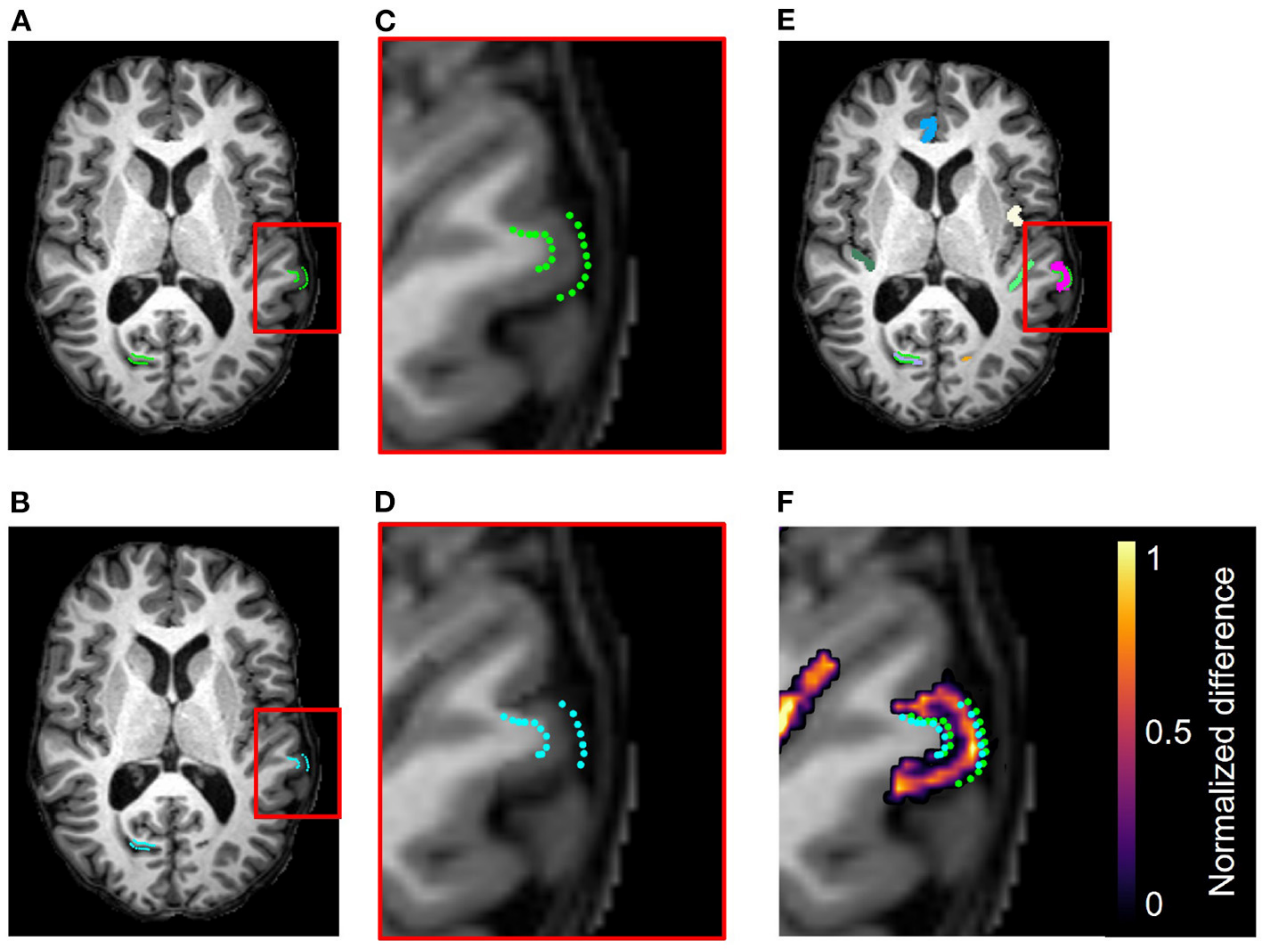
Example results from the accuracy validation for longitudinal cortical segmentation using the JHU Cortical Validation dataset and two iterations of binary morphology operations to induce atrophy. **(A)** Skull-stripped original T1w image, overlaid with original GM and WM landmarks (green). Two clusters of landmark pairs are visible in this slice: the right calcarine fissue (RCALC cluster) and the left superior temporal gyrus (LST cluster). **(B)** Skull-stripped T1w image with synthetically induced atrophy, overlaid with deformed GM and WM landmarks (blue). Again, the same RCALC and LST landmark clusters are visible in this slice. **(C)** Close-up of LST ROI selected for deformation and its landmark cluster. **(D)** Close-up of deformed LST ROI and landmark cluster. **(E)** Multi-color label map overlayed onto original T1w image, with the LST ROI in pink. **(F)** Difference image thresholded to display only non-zero voxels, as well as the original and deformed landmarks, overlaid on the original image. Note that other deformed ROIs can also be observed in this panel that are associated with additional clusters of landmarks, but that those sets are not visible in this slice.

Figure 6 displays the atrophy amount in each ROI within the DK atlas projected onto the cortical surface (Figure 6E) for several different kernel sizes. Specifically, Figure 6A displays the mean original thickness values for each region, while Figures 6B–D show the mean change in CT for *k* = 2 (used to produce the data in Figure 7), *k* = 4 (used to produce the data in Figure 4), and *k* = 12, respectively. These kernel sizes correspond to approximately 0.9 mm, 1.8 mm, and 5.5 mm of intended thickness change. We observe that for *k* = 2 (Figure 6B), there exists roughly a 0.6 mm change in thickness across the entire cortex with little variation between regions. As *k* increases, more disparities in the atrophy amount occur between ROIs consistent with their original thickness. In other words, the higher the original thickness, the more iterations we can keep applying synthetic atrophy before we reach the limit. This data is also displayed numerically in Supplementary Table 1, along with the SCPD thicknesses of each ROI in the original and maximally atrophied (*k* = 12) timepoints.

### 4.3. Qualitative Evaluation of Cortical Validation Data

Figure 7 illustrates, in addition to the conclusions stated in Section 4.1, that the deformation field can be used to translate the landmarks from their original position to the new GM/CSF interface after synthetic atrophy. In this example, we used an effective erosion kernel of *k* = 2 (2 × 2 × 2 voxels), corresponding to 2 iterations of binary morphology operations, to compute the synthetic atrophy deformation field. The original and deformed sets of left superior temporal (LST) landmarks are visible within the difference image in Figure 7F. We note that the number of visible landmarks decreases between Figures 7A–D because the deformation field displaces the fiducials in all 3 dimensions, thus moving some landmarks to a different axial slice.

### 4.4. Effect on Longitudinal Cortical Segmentation Accuracy

**Figure 8:**
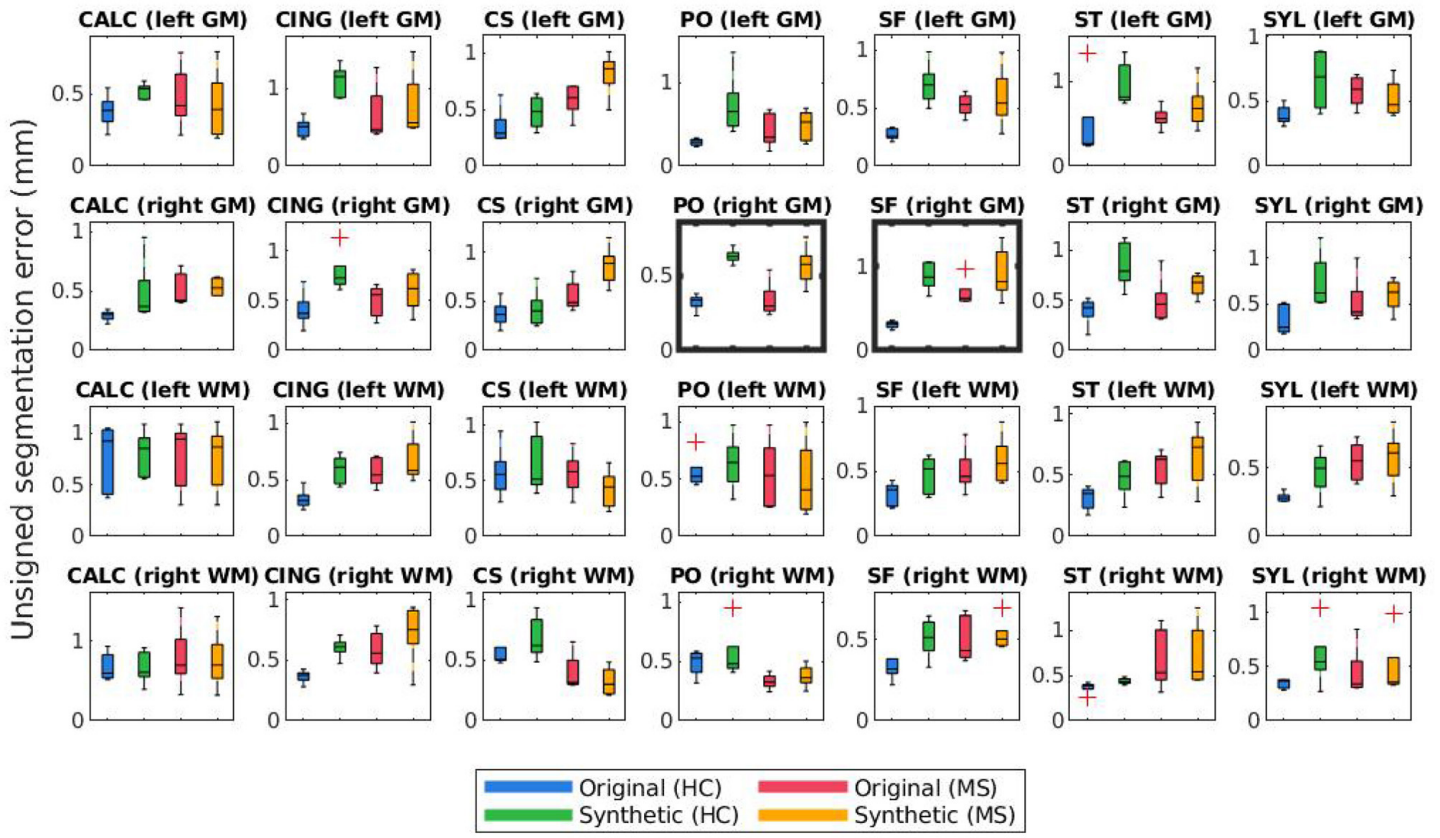
Mean unsigned segmentation errors of longitudinal FreeSurfer cortical surface reconstructions for each set of landmarks placed by expert A. Subplot rows correspond to surface and hemisphere while the subplot columns correspond to the landmarks' anatomical placements. Within each subplot, mean segmentation errors from the original images of healthy subjects are shown in blue, synthetic images of healthy subjects in green, original images of MS subjects in red, and synthetic images from MS subjects in yellow. A panel outlined in bold indicates that the statistical analysis of the associated data yielded at least one significant difference between the four groups.

**Figure 9:**
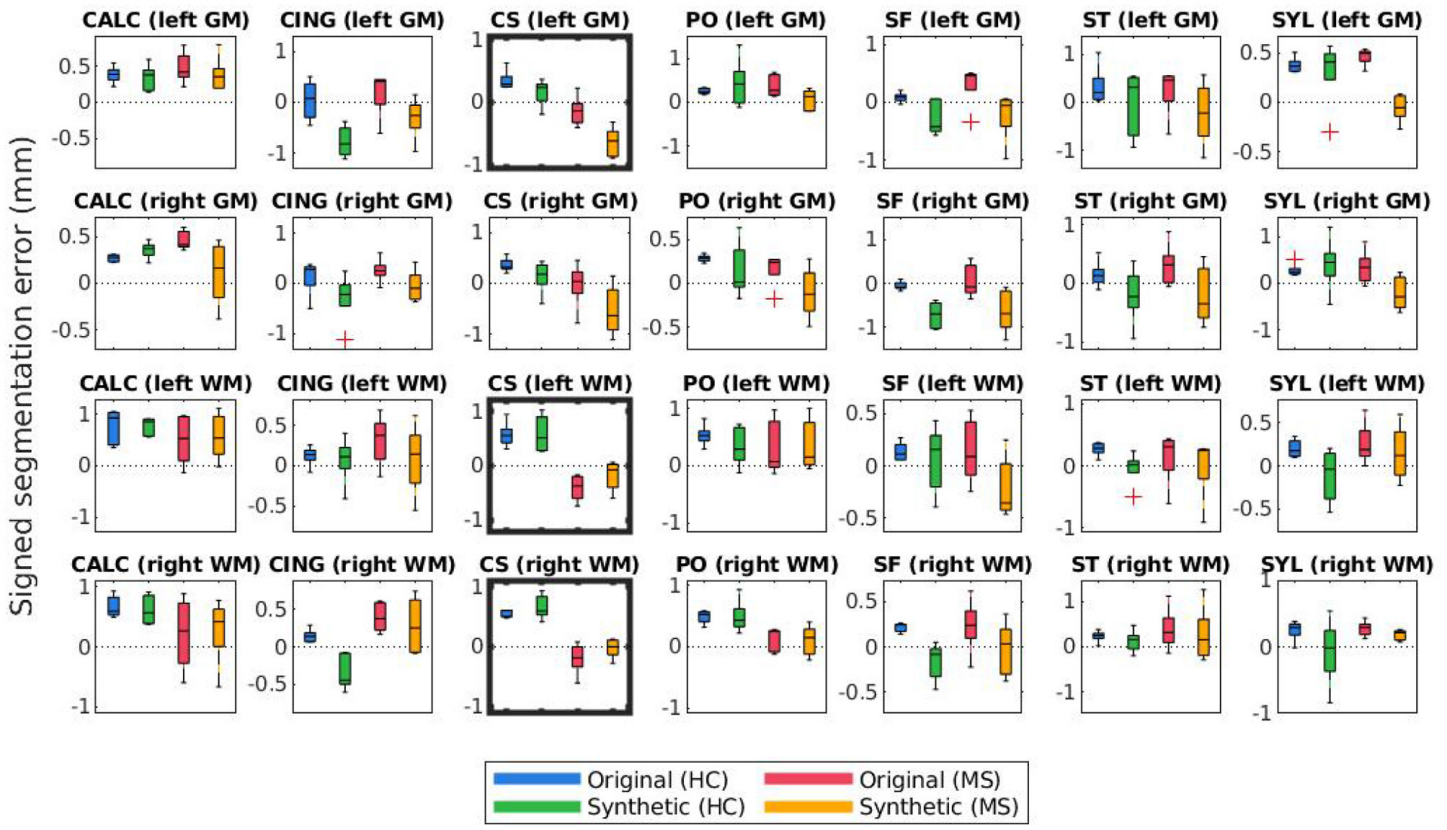
Mean signed segmentation errors of longitudinal FreeSurfer cortical surface reconstructions for each set of landmarks placed by expert A. Subplot rows correspond to surface and hemisphere while the subplot columns correspond to the landmarks' anatomical placements. Within each subplot, mean segmentation errors from the original images of healthy subjects are shown in blue, synthetic images of healthy subjects in green, original images of MS subjects in red, and synthetic images from MS subjects in yellow. A panel outlined in bold indicates that the statistical analysis of the associated data yielded at least one significant difference between the four groups.

Figures 8, 9 display the mean unsigned and signed errors, respectively, for the FreeSurfer cortical surface reconstructions of the JHU cortical validation dataset. Each figure contains 28 subplots (7 ROIs × 2 hemispheres × 2 surfaces) comparing four measurements: the error for unaltered images from HC subjects (original HC), synthetically atrophied images from HC subjects (synthetic HC), unaltered images from MS subjects (original MS), and synthetically atrophied images from MS subjects (synthetic MS). Each column corresponds to the landmarks' anatomical placements and each row to the surface (GM vs. WM) and hemisphere. For brevity, only the data using the landmarks from expert A and the longitudinal processing pipeline are shown. The entire sets of unsigned and signed errors (from both raters and both the cross-sectional and longitudinal pipelines) are shown in Supplementary Tables 2, 3.

The bolded panels in Figures 8, 9 indicate the one-way ANOVA tests that yielded *p* < 0.05 after applying a Bonferroni correction of *n* = 56 (28 sets of landmarks × 2 types of errors). This analysis was also conducted on the entire dataset: first with no correction factor and then with a correction factor of *n* = 224 (2 processes (cross-sectional vs. longitudinal) × 2 experts × 28 sets of landmarks × 2 types of errors). Table 1 details the percent of significant tests for each comparison prior to Bonferroni correction. After correction, less than 1% of all relevant tests results are significant. In this context, we define “relevant” tests as those between the original HC and synthetic HC cohorts, the synthetic HC and original MS cohorts, and the original MS and synthetic MS cohorts. By default, the multiple comparisons analyses also compared the original HC and original MS cohorts, and the synthetic HC and synthetic MS cohorts. However, these are deemed irrelevant to our study because we are not investigating the HC-MS group differences, and therefore we omitted these from the data in Table 1. In summary, there were a total of 672 statistical tests (2 processes × 2 experts × 28 sets of landmarks × 3 relevant comparisons × 2 types of errors).

**Table 1:** Percent of tests resulting in significant differences for unsigned and signed errors of FreeSurfer segmentation results *before* applying Bonferroni correction for multiple comparisons.

	**HC (O) vs. HC (S)**	**HV (S) vs. MS (O)**	**MS (O) vs. MS (S)**	**Total**
Unsigned	16.96 % (19/112)	6.25 % (7/112)	2.79 % (2/112)	8.33 % (28/336)
Signed	8.93 % (10/112)	18.75 % (21/112)	8.93 % (10/112)	12.20 % (41/336)
Total	12.95 % (29/224)	12.5 % (28/672)	5.35 % (12/672)	10.27 % (69/672)

*In parenthesis are the raw number of tests with p < 0.05. A total of 336 statistical tests using a one-way ANOVA with multiple comparisons were performed for each type of error (2 processes × 2 experts × 28 landmarks × 3 columns below). In the column titles, (O) denotes the original timepoint, and (S) the synthetic*.

**Figure 10:**
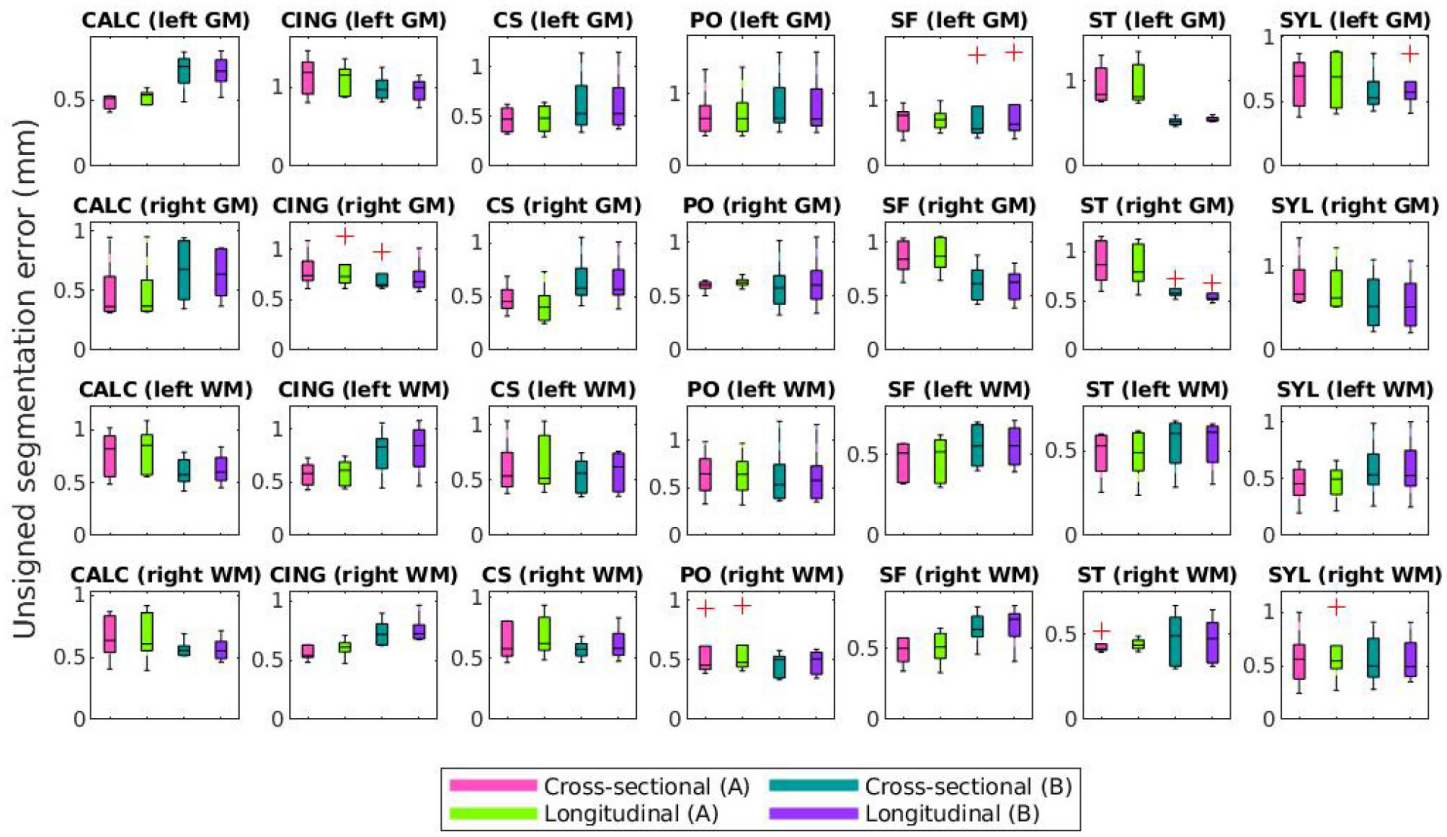
Mean unsigned segmentation errors of FreeSurfer cortical surface reconstructions of the synthetic healthy control cohort. Subplot rows correspond to surface and hemisphere while the subplot columns correspond to the landmarks' anatomical placements. Within each subplot, mean errors from the cross-sectional pipeline and measured with landmarks from expert A are in purple, the longitudinal pipeline with expert A in teal, cross-sectional with expert B in green, and longitudinal with expert B in pink. A panel outlined in bold indicates that the statistical analysis of the associated data yielded at least one significant difference between the four groups (and no bolded panels indicates no significant differences detected).

**Figure 11:**
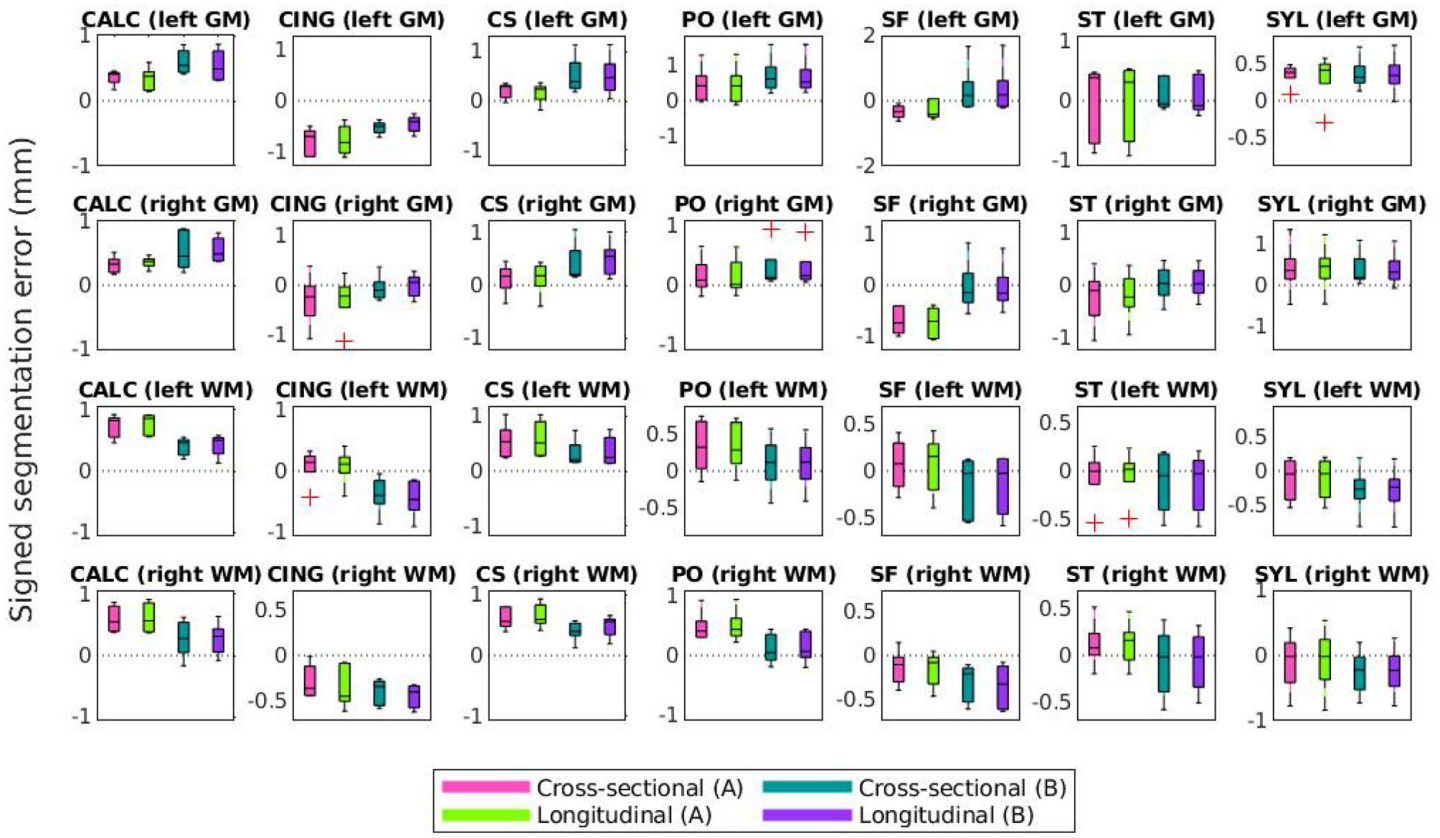
Mean signed segmentation errors of FreeSurfer cortical surface reconstructions of the synthetic healthy control cohort. Subplot rows correspond to surface and hemisphere while the subplot columns correspond to the landmarks' anatomical placements. Within each subplot, mean errors from the cross-sectional pipeline and measured with landmarks from expert A are in purple, the longitudinal pipeline with expert A in teal, cross-sectional with expert B in green, and longitudinal with expert B in pink. A panel outlined in bold indicates that the statistical analysis of the associated data yielded at least one significant difference between the four groups (and no bolded panels indicates no significant differences detected).

Lastly, Figures 10, 11 compare mean unsigned and signed errors corresponding to each expert and process type (cross-sectional vs. longitudinal FreeSurfer workflows). The row and column structures of these figures match those of Figures 8, 9. For brevity, we show only the results for the synthetic HC data. Note that the light green data bars in Figures 10, 11 show the same data as the darker green bars in Figures 8, 9. As before, we conducted similar one-way ANOVA tests for this data, also with a Bonferroni correction of *n* = 56. We found that no significant differences exist between segmentation errors produced by different processes or experts within the same set of images.

We note that trends identified in the data displayed in Figures 8–11 represent those present in the full dataset. However, because each figure includes only one out of four total configurations, we include the entire set of results in the Supplementary Materials (Supplementary Figures 1–4). Clustering the data in these two alternative formats (Figures 8, 9 and Supplementary Figures 1, 2 vs. that of Figures 10, 11 and Supplementary Figures 3, 4) allows the reader to more easily observe trends across different methods and data types. Again, this data is also shown in Supplementary Tables 2, 3.

## 5. Discussion

### 5.1. Qualitative Evaluation of Atrophied Images and Cortical Validation Data

The synthetic images resulting from our synthetic atrophy pipeline, shown in Figures 4, 7, appear visually plausible compared to the original anatomical data. The FLAIR images and fiducial landmarks both deform in a way that matches their corresponding T1w images, as expected. These results also indicate that the presented methods can induce atrophy within a desired region without affecting the surrounding tissue. The T1w difference images in Figures 4J, 7F show that the two timepoints are identical outside the ROI, as intended.

Although Figures 4, 7 indicate our pipeline performs as intended, it is worth noting that they do not necessarily depict cortical atrophy as it would appear naturally. Healthy aging and most neurodegenerative pathologies are often associated with changes in tissue appearance in addition to purely geometrical changes, and often a degree of both GM and WM atrophy are observed together rather than GM atrophy in isolation. We acknowledge that, because our methods induce changes only to the GM, we are generating only an approximation of cortical atrophy. However, our goal in this work is not to create a realistic representation of naturally occurring cortical changes, but to develop a tool used specifically for accuracy validation of cortical segmentation and thickness measurement. We specifically designed our morphology-based atrophy induction to preserve the GM/WM interface so that only the GM/CSF boundary would deform. Geometrically, the WM surface is much less complex than the GM and its segmentation is therefore an easier task. Moreover, ensuring that we alter only one of the two boundaries required for measuring CT changes simplifies the problem compared to if both boundaries were significantly deformed between timepoints.

**Figure 12:**
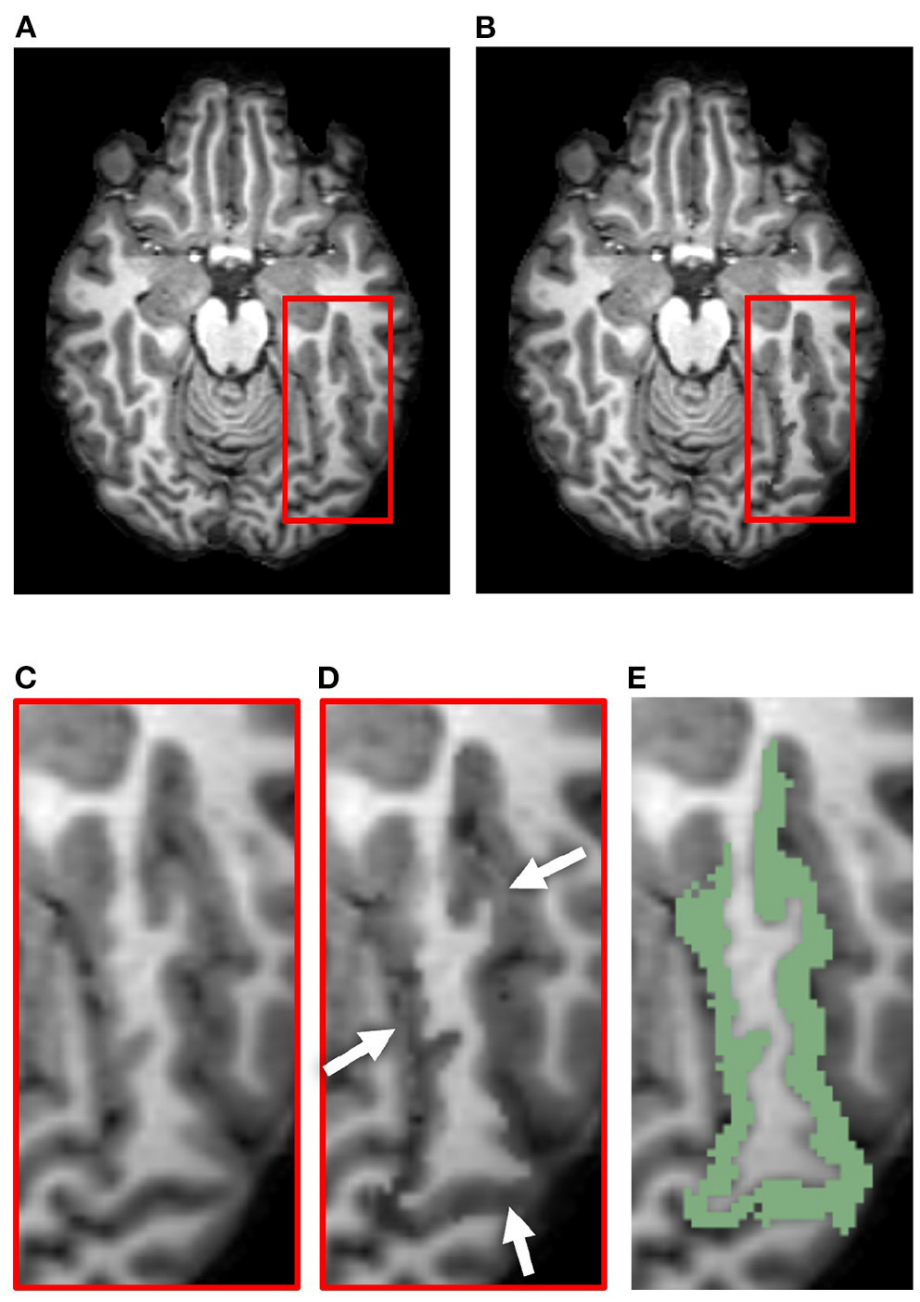
Example of less desirable results from our synthetic atrophy pipeline, where the GM surrounding the ROI is expanded in the absence of visible CSF. **(A)** Skull-stripped original T1w image. **(B)** Skull-stripped T1w image with synthetically induced atrophy. **(C)** Close-up of ROI selected for deformation. **(D)** Close-up of atrophied ROI. Arrows point to places in the image where the GM from surrounding gyri is deformed instead of CSF. **(E)** Skull-stripped original T1w image with ROI (left fusiform gyrus) overlayed in green.

When testing our method in various ROIs within the DK atlas, we found that our method performs best when operating upon a cortical ROI adjacent to CSF clearly visible within the image, such as that within a wide sulcus or the sub-arachnoid space. Because of this, the LSTG proved a perfect example. When inducing atrophy on a single side of a tight sulcus, we found that our pipeline yielded less desirable results. This is because the deformation simultaneously compresses the GM layer and expands the neighboring CSF; however, if there exists no visible CSF in voxels bordering the GM of the ROI, then it simply expands the GM on the other bank of the sulcus. An example of this is shown in Figure 12, which displays the synthetic atrophy results in the left fusiform gyrus with four iterations of binary operations to induce atrophy (the same amount as in Figure 4). The sulci surrounding this ROI are too narrow and are prone to partial volume effects; because of this, the algorithm cannot expand the CSF within this region but rather fuses the GM from opposing banks of the sulcus. We thus see an artificial increase in CT in parts of the ROI, rather than the intended decrease. Future work to address this issue may involve synthetically inserting CSF voxels to improve the quality of the deformation within such regions.

### 5.2. Localization and Extent of Cortical Atrophy

Figure 5 confirms that, as expected, increasing the effective erosion kernel size with more iterations of binary morphology operations yields a larger change in CT. Because we use high resolution ROIs to produce the deformation, we are able to induce atrophy on a sub-voxel scale; the change in cortical thickness can thus be less than the resolution of the original images. Further, the thicknesses at vertices outside the ROI boundary remain stable. This supports what we qualitatively observed in the difference images within Figures 4F, 7F: the tissue outside the ROI remains unchanged.

In Figure 6B, we observe that the limit of atrophy varies between regions but is consistent within corresponding ROIs across hemispheres. That is because this limit is determined by the original thickness of each ROI, as well as the regularization term in the deformable registration, which may not allow for a total collapse of the GM ribbon. This threshold amount—the upper extent of the atrophy we were able to induce within each region—is displayed in Figure 6D. For reference, a map of the regions studied in the analysis (Figure 6E) and the SCPD thickness in each region of the original timepoint (Figure 6A) are also included. We observe that we can induce cortical thinning up to between 0.8 and 2.5 mm, which translates to approximately between 40% and 80% of a region's original thickness (as measured with the SCPD formula).

The most challenging aspect of establishing ground truth is avoiding bias induced by the methods employed for its measurement. For this reason, we elected to use the marching-cubes algorithm to obtain surface representations rather than a more thoroughly validated pipeline such as FreeSurfer or CRUISE (Han et al., 2004). When applying these methods for the quantification of a new method for CT measurement, one could create the synthetic atrophy dataset with our morphology-based methods, generate surface representations of each timepoint using an established segmentation pipeline, and then return back to our method to assess true change using those surfaces rather than ones obtained with marching cubes. In this case, the ground truth measurements would indeed be biased by the selected cortical segmentation method, but would use surfaces that better represent the complex geometry of cortical GM. That being said, the advantage to using marching cubes surfaces is that the resulting surface placement corresponds exactly with GM/WM and GM/CSF boundaries of the ROIs selected for deformation. The use of an established cortical segmentation pipeline may introduce additional discrepancies between the ground truth and measured thickness changes.

We also acknowledge that our definition of the true change in thickness differs from the traditional definition of CT. In longitudinal studies of CT, thickness change is defined as the difference between thickness measured at each timepoint. However, we define this change as the difference in surface placement. Had we elected to define thickness change in the traditional way, our results would have been biased by whatever method we employed to measure CT. We circumvent this problem by removing the actual thickness calculation from the pipeline, and simply find the amount by which the GM and WM surfaces have been displaced by the synthetic atrophy deformation.

Finally, although the methods discussed for inducing and assessing localized cortical atrophy are presented in this paper as a single pipeline, they are not dependent upon each other. For example, one could apply the methods presented here to obtain a synthetic longitudinal dataset with a transformation encoding the amount to which each image has been atrophied, and measure CT volumetrically rather than with cortical surfaces. Alternatively, one could employ a different technique to synthetically induce atrophy, such as that proposed by Karaçali and Davatzikos (2006), obtain cortical surfaces corresponding to the original and synthetic timepoints, and then apply our method to assess the true change in CT. Further, our methods are not restricted to using FreeSurfer to obtain a cortical parcellation and skull-strip mask; any parcellation that contains separate labels for GM, WM, and CSF will suffice, as well as any skull-strip mask.

### 5.3. Effect on Longitudinal Cortical Segmentation Accuracy

Figures 8, 9 illustrate the unsigned and signed errors between timepoints (original vs. atrophy). HC and MS subjects were analyzed separately rather than as a single cohort to illustrate that FreeSurfer yields surfaces with higher errors for both natural and synthetic atrophy, rather than exclusively for the synthetic atrophy induced by our methods. We observe that in general, for HC subjects, FreeSurfer has lower errors for the original timepoints than the synthetic. This may lead one to prematurely conclude that the FreeSurfer produces erroneous segmentations when processing images created by the proposed methods, perhaps due to slight blurring that results from interpolation. However, when compared to the original timepoint for the MS subjects, there exist no significant differences between the HC-synthetic (synthetic atrophy) and MS-original (natural atrophy) cohorts based on our corrected multiple comparisons analysis. Thus, it follows that these errors likely arise from the presence of any atrophy at all, rather than whether or not the image is synthetic. This is not an unexpected finding about FreeSurfer, as many algorithms have a decrease in performance as the data deviates from healthy controls. We note that the exception to this is within the right WM central sulcus cluster; the absolute signed errors are similar, but they have opposite signs, which indicates that in this location, FreeSurfer is overestimating surface placement in the HC images while underestimating the MS.

Figures 10, 11 show that within the healthy synthetic cohort, there exist only slight differences in segmentation errors between experts, none of them statistically significant. We observe even smaller differences between images processed cross-sectionally vs. longitudinally. These results further support our previous conclusion that the data shown in Figures 8, 9 are indeed suitable representations of the entire set. Further, the lack of discrepancy between results from the cross-sectional and longitudinal processing methods show that the inclusion of synthetic data in FreeSurfer's joint initialization steps (Reuter et al., 2012) does not corrupt the final longitudinal segmentation results.

Table 1 shows that within the entire dataset (not simply those shown in Figures 8–11), there exists a much larger number of statistically significant mean signed segmentation errors than unsigned. This implies that FreeSurfer is overestimating surface placement in some places while underestimating in others. After applying a Bonferroni correction of *n* = 224, we found only 14 out of all 1,344 tests yielded significant differences, and only 3 out of the 672 deemed “relevant”. These errors are almost entirely limited to within the central sulcus (shown in the three bolded panels within Figure 9, and may be due to its higher than average thickness compared to the rest of the brain. Further, these instances, the significant differences exist between the HC and MS groups as a whole, rather than simply between the synthetic HC and original MS cohorts, which suggests the segmentation errors may arise from the original landmark placement or image quality rather than due to errors induced by our methods. Overall, these results support our observation that FreeSurfer performs somewhat worse in all cases of atrophy, regardless of whether it is synthetic or natural.

Lastly, we found that the success of our methods in this context is also dependent on fiducial landmark placement. For example, the central sulcus landmark set for one subject in the HC dataset contained two clusters in adjacent slices where one cluster existed directly on top of the other in an adjacent slice. This means that, rather then both ROIs having 3 slices of padding on either side of the landmark cluster, each ROI had 3 slices of padding on one side and none on the other. Further, in order to induce atrophy in both ROIs, the image was warped in slightly different directions in adjacent slices, so deformations at the cluster locations could have been affected by boundary effects due to the lack of padding. All this could have potentially induced image artifacts that hindered FreeSurfer's ability to yield accurate segmentations. This could be addressed by fine tuning the landmark locations such that each ROI would include an adequate buffer around the fiducials. Alternatively, instead of deforming each individual cluster separately, certain landmark clusters could be combined into a single, larger cluster and deformed as a single ROI, which would remove the issue of adjacent slices being deformed by discontinuous transformations. Our method proved to perform best when operating on isolated landmark sets rather than those placed close together.

## 6. Conclusion

In summary, we presented a registration-based method for inducing synthetic, localized cortical atrophy in MRI scans. The quantitative evaluations illustrate that this technique can be used for accuracy validation of CT measurements, specifically those obtained using surface-based methods, by comparing experimentally measured values to the ground truth produced by our algorithm. Further, we showed that our work is also applicable to accuracy validation of cortical segmentation pipelines; the methods can be used to produce a set of longitudinal cortical landmarks with exact correspondences between the original and atrophied timepoints. Importantly, our method relies exclusively on publicly available software and datasets.

## Data Availability Statement

All code custom-written for this pipeline is publicly available on Github. This data can be found here: https://github.com/MedICL-VU/Synthetic-Atrophy-For-Longtidunal-Cortical-Surface-Analyses.

## Ethics Statement

Ethical review and approval was not required for the study on human participants in accordance with the local legislation and institutional requirements. Written informed consent for participation was not required for this study in accordance with the national legislation and the institutional requirements.

## Author Contributions

KL contributed the majority of the algorithm development, data processing and analysis, and manuscript writing, with ample consultation with IO. Both authors conceived the presented ideas and theories in this manuscript.

## Funding

This work was supported in part, by NIH grant R01-NS094456.

## Conflict of Interest

The authors declare that the research was conducted in the absence of any commercial or financial relationships that could be construed as a potential conflict of interest.

## Publisher's Note

All claims expressed in this article are solely those of the authors and do not necessarily represent those of their affiliated organizations, or those of the publisher, the editors and the reviewers. Any product that may be evaluated in this article, or claim that may be made by its manufacturer, is not guaranteed or endorsed by the publisher.

## Footnotes

^1^ http://iacl.ece.jhu.edu/index.php?title=Cortical_data/

^2^ https://itk.org/

^3^ https://www.slicer.org/wiki/Documentation/4.3/Modules/ModelMaker

^4^ https://vtk.org/

^5^ https://slicer.readthedocs.io/en/latest/user_guide/modules/segmenteditor.html
