# Supplementary material for: Synthetic Atrophy for Longitudinal Cortical Surface Analyses
Source: Front Neuroimaging. 2022 Jun 2;1:861687. doi: 10.3389/fnimg.2022.861687 (PMC10406236; doi:10.3389/fnimg.2022.861687)
Supplement: Supplementary file 1 [file Data_Sheet_1.PDF]

Supplementary Material

Table S1. Synthetic atrophy limits for each ROI selected from the DK atlas. True change in thickness is measured using the mean surface displacement difference (MSDD). This data is also displayed as a cortical parcellation onto an example marching cubes surface in Figure 6. These results are shown here alongside the original thickness and maximum change in thickness measured using the SCP distance formula.

FreeSurfer Cortical Label	Mean original SCP thickness (mm)	Mean SCP thickness at atrophy limit (mm)	Mean MSDD thickness change at atrophy limit (mm)
lh-caudalanteriorcingulate	2.88 ± 0.20	1.43 ± 0.20	1.34 ± 0.23
rh-caudalanteriorcingulate	2.72 ± 0.22	1.24 ± 0.23	1.21 ± 0.26
lh-caudalmiddlefrontal	2.90 ± 0.12	1.90 ± 0.14	1.75 ± 0.15
rh-caudalmiddlefrontal	2.91 ± 0.17	1.82 ± 0.16	1.85 ± 0.17
lh-cuneus	2.11 ± 0.14	1.17 ± 0.10	1.16 ± 0.10
rh-cuneus	2.05 ± 0.12	1.12 ± 0.11	1.10 ± 0.10
lh-entorhinal	2.26 ± 0.22	1.47 ± 0.23	1.63 ± 0.16
rh-entorhinal	2.35 ± 0.24	1.41 ± 0.21	2.07 ± 0.31
lh-fusiform	3.06 ± 0.17	2.24 ± 0.17	2.32 ± 0.16
rh-fusiform	3.06 ± 0.18	2.22 ± 0.14	2.42 ± 0.16
lh-inferiorparietal	2.86 ± 0.10	1.90 ± 0.09	1.87 ± 0.11
rh-inferiorparietal	2.90 ± 0.10	1.90 ± 0.09	1.89 ± 0.14
lh-inferiortemporal	3.18 ± 0.16	2.22 ± 0.12	2.26 ± 0.11
rh-inferiortemporal	3.16 ± 0.19	2.09 ± 0.15	2.36 ± 0.15
lh-isthmuscingulate	2.48 ± 0.13	1.13 ± 0.21	1.30 ± 0.18
rh-isthmuscingulate	2.45 ± 0.16	1.06 ± 0.25	1.25 ± 0.21
lh-lateraloccipital	2.43 ± 0.14	1.68 ± 0.13	1.82 ± 0.13
rh-lateraloccipital	2.51 ± 0.14	1.74 ± 0.11	1.86 ± 0.14
lh-lateralorbitofrontal	2.70 ± 0.16	1.82 ± 0.12	1.98 ± 0.12
rh-lateralorbitofrontal	2.72 ± 0.14	1.84 ± 0.11	2.00 ± 0.10
lh-lingual	2.18 ± 0.19	1.43 ± 0.18	1.62 ± 0.15
rh-lingual	2.21 ± 0.13	1.47 ± 0.12	1.61 ± 0.14
lh-medialorbitofrontal	2.55 ± 0.15	1.28 ± 0.36	1.31 ± 0.37
rh-medialorbitofrontal	2.56 ± 0.22	1.30 ± 0.15	1.33 ± 0.14
lh-middletemporal	3.27 ± 0.13	2.18 ± 0.12	2.11 ± 0.14
rh-middletemporal	3.27 ± 0.18	2.07 ± 0.14	2.14 ± 0.10
lh-parahippocampal	2.44 ± 0.19	1.78 ± 0.13	1.80 ± 0.19
rh-parahippocampal	2.42 ± 0.23	1.69 ± 0.22	1.80 ± 0.20
lh-paracentral	2.84 ± 0.12	1.70 ± 0.11	1.60 ± 0.20
rh-paracentral	2.83 ± 0.19	1.58 ± 0.60	1.67 ± 0.27
lh-parsopercularis	2.91 ± 0.16	1.84 ± 0.14	1.65 ± 0.12
rh-parsopercularis	2.96 ± 0.18	1.85 ± 0.14	1.63 ± 0.15
lh-parsorbitalis	2.98 ± 0.18	1.91 ± 0.16	1.72 ± 0.14
rh-parsorbitalis	3.07 ± 0.17	1.99 ± 0.12	1.76 ± 0.09
lh-parstriangularis	2.98 ± 0.13	2.01 ± 0.11	1.84 ± 0.13
rh-parstriangularis	2.98 ± 0.16	1.95 ± 0.14	1.81 ± 0.17
lh-pericalcarine	1.67 ± 0.17	0.73 ± 0.18	0.76 ± 0.20
rh-pericalcarine	1.80 ± 0.12	0.84 ± 0.08	0.77 ± 0.18
lh-postcentral	2.34 ± 0.15	1.42 ± 0.11	1.37 ± 0.14
rh-postcentral	2.27 ± 0.12	1.35 ± 0.11	1.33 ± 0.10
lh-posteriorcingulate	2.65 ± 0.15	1.06 ± 0.19	1.12 ± 0.24
rh-posteriorcingulate	2.66 ± 0.09	1.03 ± 0.17	1.10 ± 0.16
lh-precentral	2.81 ± 0.13	1.76 ± 0.08	1.77 ± 0.11
rh-precentral	2.81 ± 0.15	1.77 ± 0.10	1.83 ± 0.12
lh-precuneus	2.73 ± 0.14	1.56 ± 0.15	1.47 ± 0.14
rh-precuneus	2.66 ± 0.14	1.48 ± 0.13	1.39 ± 0.13
lh-rostralanteriorcingulate	2.80 ± 0.20	1.06 ± 0.13	1.00 ± 0.13
rh-rostralanteriorcingulate	2.68 ± 0.23	0.93 ± 0.22	0.97 ± 0.20
lh-rostralmiddlefrontal	2.87 ± 0.12	1.90 ± 0.10	1.89 ± 0.13
rh-rostralmiddlefrontal	2.90 ± 0.14	1.91 ± 0.12	1.92 ± 0.15
lh-superiorfrontal	3.05 ± 0.14	1.95 ± 0.10	1.97 ± 0.11
rh-superiorfrontal	3.05 ± 0.15	1.92 ± 0.11	1.99 ± 0.11
lh-superiorparietal	2.54 ± 0.12	1.61 ± 0.10	1.54 ± 0.11
rh-superiorparietal	2.51 ± 0.13	1.57 ± 0.12	1.52 ± 0.12
lh-superiortemporal	3.17 ± 0.17	2.05 ± 0.12	2.07 ± 0.16
rh-superiortemporal	3.15 ± 0.23	1.98 ± 0.18	2.07 ± 0.13
lh-supramarginal	2.88 ± 0.16	1.88 ± 0.12	1.78 ± 0.14
rh-supramarginal	2.82 ± 0.20	1.82 ± 0.14	1.72 ± 0.17
lh-transversetemporal	2.26 ± 0.37	1.22 ± 0.29	1.12 ± 0.21
rh-transversetemporal	2.27 ± 0.39	1.10 ± 0.21	0.99 ± 0.24
lh-insula	3.27 ± 0.14	1.98 ± 0.10	2.05 ± 0.18
rh-insula	3.26 ± 0.24	1.97 ± 0.17	2.09 ± 0.23

Table S2. Mean unsigned segmentation errors of FreeSurfer cortical surface reconstructions for each set of landmarks across healthy and MS subjects.

		Left GM				Right GM			
		**3D (A)**	**4D (A)**	**3D (B)**	**4D(B)**	**3D (A)**	**4D (A)**	**3D (B)**	**4D(B)**
**CALC**	**HC (0)**	0.37 ± 0.09	0.37 ± 0.09	0.37 ± 0.09	0.37 ± 0.09	0.27 ± 0.04	0.27 ± 0.04	0.27 ± 0.04	0.27 ± 0.04
	**HC (1)**	0.48 ± 0.05	0.48 ± 0.05	0.48 ± 0.05	0.48 ± 0.05	0.49 ± 0.24	0.49 ± 0.24	0.49 ± 0.24	0.49 ± 0.24
	**MS (0)**	0.49 ± 0.15	0.49 ± 0.15	0.49 ± 0.15	0.49 ± 0.15	0.51 ± 0.16	0.51 ± 0.16	0.51 ± 0.16	0.51 ± 0.16
	**MS (1)**	0.48 ± 0.18	0.48 ± 0.18	0.48 ± 0.18	0.48 ± 0.18	0.57 ± 0.10	0.57 ± 0.10	0.57 ± 0.10	0.57 ± 0.10
**CING**	**HC (0)**	0.76 ± 0.27	0.76 ± 0.27	0.76 ± 0.27	0.76 ± 0.27	0.63 ± 0.16	0.63 ± 0.16	0.63 ± 0.16	0.63 ± 0.16
	**HC (1)**	0.77 ± 0.20	0.77 ± 0.20	0.77 ± 0.20	0.77 ± 0.20	0.66 ± 0.17	0.66 ± 0.17	0.66 ± 0.17	0.66 ± 0.17
	**MS (0)**	1.11 ± 0.64	1.11 ± 0.64	1.11 ± 0.64	1.11 ± 0.64	1.02 ± 0.73	1.02 ± 0.73	1.02 ± 0.73	1.02 ± 0.73
	**MS (1)**	0.71 ± 0.28	0.71 ± 0.28	0.71 ± 0.28	0.71 ± 0.28	0.91 ± 0.62	0.91 ± 0.62	0.91 ± 0.62	0.91 ± 0.62
**CS**	**HC (0)**	0.47 ± 0.13	0.47 ± 0.13	0.47 ± 0.13	0.47 ± 0.13	0.39 ± 0.17	0.39 ± 0.17	0.39 ± 0.17	0.39 ± 0.17
	**HC (1)**	1.13 ± 0.23	1.13 ± 0.23	1.13 ± 0.23	1.13 ± 0.23	0.79 ± 0.16	0.79 ± 0.16	0.79 ± 0.16	0.79 ± 0.16
	**MS (0)**	0.53 ± 0.37	0.53 ± 0.37	0.53 ± 0.37	0.53 ± 0.37	0.48 ± 0.11	0.48 ± 0.11	0.48 ± 0.11	0.48 ± 0.11
	**MS (1)**	0.79 ± 0.39	0.79 ± 0.39	0.79 ± 0.39	0.79 ± 0.39	0.55 ± 0.16	0.55 ± 0.16	0.55 ± 0.16	0.55 ± 0.16
**PO**	**HC (0)**	0.32 ± 0.08	0.32 ± 0.08	0.32 ± 0.08	0.32 ± 0.08	0.37 ± 0.04	0.37 ± 0.04	0.37 ± 0.04	0.37 ± 0.04
	**HC (1)**	0.57 ± 0.11	0.57 ± 0.11	0.57 ± 0.11	0.57 ± 0.11	0.56 ± 0.06	0.56 ± 0.06	0.56 ± 0.06	0.56 ± 0.06
	**MS (0)**	0.54 ± 0.11	0.54 ± 0.11	0.54 ± 0.11	0.54 ± 0.11	0.61 ± 0.15	0.61 ± 0.15	0.61 ± 0.15	0.61 ± 0.15
	**MS (1)**	0.72 ± 0.21	0.72 ± 0.21	0.72 ± 0.21	0.72 ± 0.21	0.78 ± 0.24	0.78 ± 0.24	0.78 ± 0.24	0.78 ± 0.24
**SF**	**HC (0)**	0.35 ± 0.15	0.35 ± 0.15	0.35 ± 0.15	0.35 ± 0.15	0.37 ± 0.12	0.37 ± 0.12	0.37 ± 0.12	0.37 ± 0.12
	**HC (1)**	0.47 ± 0.12	0.47 ± 0.12	0.47 ± 0.12	0.47 ± 0.12	0.48 ± 0.12	0.48 ± 0.12	0.48 ± 0.12	0.48 ± 0.12
	**MS (0)**	0.72 ± 0.13	0.72 ± 0.13	0.72 ± 0.13	0.72 ± 0.13	0.54 ± 0.22	0.54 ± 0.22	0.54 ± 0.22	0.54 ± 0.22
	**MS (1)**	0.91 ± 0.19	0.91 ± 0.19	0.91 ± 0.19	0.91 ± 0.19	0.81 ± 0.19	0.81 ± 0.19	0.81 ± 0.19	0.81 ± 0.19
**ST**	**HC (0)**	0.58 ± 0.23	0.58 ± 0.23	0.58 ± 0.23	0.58 ± 0.23	0.54 ± 0.05	0.54 ± 0.05	0.54 ± 0.05	0.54 ± 0.05
	**HC (1)**	0.61 ± 0.23	0.61 ± 0.23	0.61 ± 0.23	0.61 ± 0.23	0.64 ± 0.14	0.64 ± 0.14	0.64 ± 0.14	0.64 ± 0.14
	**MS (0)**	0.53 ± 0.15	0.53 ± 0.15	0.53 ± 0.15	0.53 ± 0.15	0.39 ± 0.08	0.39 ± 0.08	0.39 ± 0.08	0.39 ± 0.08
	**MS (1)**	0.32 ± 0.15	0.32 ± 0.15	0.32 ± 0.15	0.32 ± 0.15	0.31 ± 0.12	0.31 ± 0.12	0.31 ± 0.12	0.31 ± 0.12
**SYL**	**HC (0)**	0.30 ± 0.03	0.30 ± 0.03	0.30 ± 0.03	0.30 ± 0.03	0.30 ± 0.04	0.30 ± 0.04	0.30 ± 0.04	0.30 ± 0.04
	**HC (1)**	0.72 ± 0.32	0.72 ± 0.32	0.72 ± 0.32	0.72 ± 0.32	0.60 ± 0.05	0.60 ± 0.05	0.60 ± 0.05	0.60 ± 0.05
	**MS (0)**	0.46 ± 0.08	0.46 ± 0.08	0.46 ± 0.08	0.46 ± 0.08	0.34 ± 0.08	0.34 ± 0.08	0.34 ± 0.08	0.34 ± 0.08
	**MS (1)**	0.61 ± 0.23	0.61 ± 0.23	0.61 ± 0.23	0.61 ± 0.23	0.62 ± 0.15	0.62 ± 0.15	0.62 ± 0.15	0.62 ± 0.15
		**Left WM**				**Right WM**			
		**3D (A)**	**4D (A)**	**3D (B)**	**4D(B)**	**3D (A)**	**4D (A)**	**3D (B)**	**4D(B)**
**CALC**	**HC (0)**	0.56 ± 0.13	0.56 ± 0.13	0.56 ± 0.13	0.56 ± 0.13	0.48 ± 0.09	0.48 ± 0.09	0.48 ± 0.09	0.48 ± 0.09
	**HC (1)**	0.65 ± 0.22	0.65 ± 0.22	0.65 ± 0.22	0.65 ± 0.22	0.54 ± 0.20	0.54 ± 0.20	0.54 ± 0.20	0.54 ± 0.20
	**MS (0)**	0.57 ± 0.30	0.57 ± 0.30	0.57 ± 0.30	0.57 ± 0.30	0.34 ± 0.07	0.34 ± 0.07	0.34 ± 0.07	0.34 ± 0.07
	**MS (1)**	0.49 ± 0.31	0.49 ± 0.31	0.49 ± 0.31	0.49 ± 0.31	0.42 ± 0.11	0.42 ± 0.11	0.42 ± 0.11	0.42 ± 0.11
**CING**	**HC (0)**	0.27 ± 0.03	0.27 ± 0.03	0.27 ± 0.03	0.27 ± 0.03	0.30 ± 0.05	0.30 ± 0.05	0.30 ± 0.05	0.30 ± 0.05
	**HC (1)**	0.69 ± 0.19	0.69 ± 0.19	0.69 ± 0.19	0.69 ± 0.19	0.86 ± 0.15	0.86 ± 0.15	0.86 ± 0.15	0.86 ± 0.15
	**MS (0)**	0.55 ± 0.13	0.55 ± 0.13	0.55 ± 0.13	0.55 ± 0.13	0.63 ± 0.14	0.63 ± 0.14	0.63 ± 0.14	0.63 ± 0.14
	**MS (1)**	0.65 ± 0.17	0.65 ± 0.17	0.65 ± 0.17	0.65 ± 0.17	0.89 ± 0.21	0.89 ± 0.21	0.89 ± 0.21	0.89 ± 0.21
**CS**	**HC (0)**	0.32 ± 0.10	0.32 ± 0.10	0.32 ± 0.10	0.32 ± 0.10	0.32 ± 0.06	0.32 ± 0.06	0.32 ± 0.06	0.32 ± 0.06
	**HC (1)**	0.46 ± 0.11	0.46 ± 0.11	0.46 ± 0.11	0.46 ± 0.11	0.49 ± 0.09	0.49 ± 0.09	0.49 ± 0.09	0.49 ± 0.09
	**MS (0)**	0.50 ± 0.11	0.50 ± 0.11	0.50 ± 0.11	0.50 ± 0.11	0.53 ± 0.12	0.53 ± 0.12	0.53 ± 0.12	0.53 ± 0.12
	**MS (1)**	0.58 ± 0.15	0.58 ± 0.15	0.58 ± 0.15	0.58 ± 0.15	0.59 ± 0.09	0.59 ± 0.09	0.59 ± 0.09	0.59 ± 0.09
**PO**	**HC (0)**	0.48 ± 0.40	0.48 ± 0.40	0.48 ± 0.40	0.48 ± 0.40	0.38 ± 0.11	0.38 ± 0.11	0.38 ± 0.11	0.38 ± 0.11
	**HC (1)**	0.96 ± 0.21	0.96 ± 0.21	0.96 ± 0.21	0.96 ± 0.21	0.89 ± 0.21	0.89 ± 0.21	0.89 ± 0.21	0.89 ± 0.21
	**MS (0)**	0.60 ± 0.15	0.60 ± 0.15	0.60 ± 0.15	0.60 ± 0.15	0.54 ± 0.23	0.54 ± 0.23	0.54 ± 0.23	0.54 ± 0.23
	**MS (1)**	0.75 ± 0.30	0.75 ± 0.30	0.75 ± 0.30	0.75 ± 0.30	0.57 ± 0.16	0.57 ± 0.16	0.57 ± 0.16	0.57 ± 0.16
**SF**	**HC (0)**	0.29 ± 0.08	0.29 ± 0.08	0.29 ± 0.08	0.29 ± 0.08	0.37 ± 0.07	0.37 ± 0.07	0.37 ± 0.07	0.37 ± 0.07
	**HC (1)**	0.48 ± 0.13	0.48 ± 0.13	0.48 ± 0.13	0.48 ± 0.13	0.43 ± 0.04	0.43 ± 0.04	0.43 ± 0.04	0.43 ± 0.04
	**MS (0)**	0.59 ± 0.17	0.59 ± 0.17	0.59 ± 0.17	0.59 ± 0.17	0.68 ± 0.26	0.68 ± 0.26	0.68 ± 0.26	0.68 ± 0.26
	**MS (1)**	0.62 ± 0.22	0.62 ± 0.22	0.62 ± 0.22	0.62 ± 0.22	0.70 ± 0.28	0.70 ± 0.28	0.70 ± 0.28	0.70 ± 0.28
**ST**	**HC (0)**	0.38 ± 0.05	0.38 ± 0.05	0.38 ± 0.05	0.38 ± 0.05	0.31 ± 0.14	0.31 ± 0.14	0.31 ± 0.14	0.31 ± 0.14
	**HC (1)**	0.64 ± 0.18	0.64 ± 0.18	0.64 ± 0.18	0.64 ± 0.18	0.80 ± 0.29	0.80 ± 0.29	0.80 ± 0.29	0.80 ± 0.29
	**MS (0)**	0.56 ± 0.11	0.56 ± 0.11	0.56 ± 0.11	0.56 ± 0.11	0.53 ± 0.25	0.53 ± 0.25	0.53 ± 0.25	0.53 ± 0.25
	**MS (1)**	0.56 ± 0.13	0.56 ± 0.13	0.56 ± 0.13	0.56 ± 0.13	0.53 ± 0.13	0.53 ± 0.13	0.53 ± 0.13	0.53 ± 0.13
**SYL**	**HC (0)**	0.28 ± 0.04	0.28 ± 0.04	0.28 ± 0.04	0.28 ± 0.04	0.34 ± 0.03	0.34 ± 0.03	0.34 ± 0.03	0.34 ± 0.03
	**HC (1)**	0.45 ± 0.15	0.45 ± 0.15	0.45 ± 0.15	0.45 ± 0.15	0.56 ± 0.25	0.56 ± 0.25	0.56 ± 0.25	0.56 ± 0.25
	**MS (0)**	0.56 ± 0.12	0.56 ± 0.12	0.56 ± 0.12	0.56 ± 0.12	0.47 ± 0.20	0.47 ± 0.20	0.47 ± 0.20	0.47 ± 0.20
	**MS (1)**	0.56 ± 0.17	0.56 ± 0.17	0.56 ± 0.17	0.56 ± 0.17	0.51 ± 0.27	0.51 ± 0.27	0.51 ± 0.27	0.51 ± 0.27

Table S3. Mean signed segmentation errors of FreeSurfer cortical surface reconstructions for each set of landmarks across healthy and MS subjects.

		Left GM				Right GM			
		**3D (A)**	**4D (A)**	**3D (B)**	**4D(B)**	**3D (A)**	**4D (A)**	**3D (B)**	**4D(B)**
**CALC**	**HC (0)**	0.37 ± 0.09	0.37 ± 0.09	0.37 ± 0.09	0.37 ± 0.09	0.25 ± 0.06	0.25 ± 0.06	0.25 ± 0.06	0.25 ± 0.06
	**HC (1)**	0.36 ± 0.10	0.36 ± 0.10	0.36 ± 0.10	0.36 ± 0.10	0.32 ± 0.12	0.32 ± 0.12	0.32 ± 0.12	0.32 ± 0.12
	**MS (0)**	0.25 ± 0.27	0.25 ± 0.27	0.25 ± 0.27	0.25 ± 0.27	0.44 ± 0.22	0.44 ± 0.22	0.44 ± 0.22	0.44 ± 0.22
	**MS (1)**	0.22 ± 0.31	0.22 ± 0.31	0.22 ± 0.31	0.22 ± 0.31	0.19 ± 0.20	0.19 ± 0.20	0.19 ± 0.20	0.19 ± 0.20
**CING**	**HC (0)**	0.72 ± 0.26	0.72 ± 0.26	0.72 ± 0.26	0.72 ± 0.26	0.62 ± 0.16	0.62 ± 0.16	0.62 ± 0.16	0.62 ± 0.16
	**HC (1)**	0.72 ± 0.17	0.72 ± 0.17	0.72 ± 0.17	0.72 ± 0.17	0.60 ± 0.20	0.60 ± 0.20	0.60 ± 0.20	0.60 ± 0.20
	**MS (0)**	0.14 ± 0.97	0.14 ± 0.97	0.14 ± 0.97	0.14 ± 0.97	-0.07 ± 0.96	-0.07 ± 0.96	-0.07 ± 0.96	-0.07 ± 0.96
	**MS (1)**	0.58 ± 0.36	0.58 ± 0.36	0.58 ± 0.36	0.58 ± 0.36	0.06 ± 0.86	0.06 ± 0.86	0.06 ± 0.86	0.06 ± 0.86
**CS**	**HC (0)**	0.04 ± 0.34	0.04 ± 0.34	0.04 ± 0.34	0.04 ± 0.34	0.06 ± 0.28	0.06 ± 0.28	0.06 ± 0.28	0.06 ± 0.28
	**HC (1)**	-0.80 ± 0.25	-0.80 ± 0.25	-0.80 ± 0.25	-0.80 ± 0.25	-0.31 ± 0.47	-0.31 ± 0.47	-0.31 ± 0.47	-0.31 ± 0.47
	**MS (0)**	0.12 ± 0.36	0.12 ± 0.36	0.12 ± 0.36	0.12 ± 0.36	0.26 ± 0.13	0.26 ± 0.13	0.26 ± 0.13	0.26 ± 0.13
	**MS (1)**	-0.41 ± 0.38	-0.41 ± 0.38	-0.41 ± 0.38	-0.41 ± 0.38	-0.02 ± 0.24	-0.02 ± 0.24	-0.02 ± 0.24	-0.02 ± 0.24
**PO**	**HC (0)**	0.12 ± 0.11	0.12 ± 0.11	0.12 ± 0.11	0.12 ± 0.11	0.13 ± 0.10	0.13 ± 0.10	0.13 ± 0.10	0.13 ± 0.10
	**HC (1)**	0.08 ± 0.26	0.08 ± 0.26	0.08 ± 0.26	0.08 ± 0.26	-0.28 ± 0.17	-0.28 ± 0.17	-0.28 ± 0.17	-0.28 ± 0.17
	**MS (0)**	0.23 ± 0.24	0.23 ± 0.24	0.23 ± 0.24	0.23 ± 0.24	0.35 ± 0.25	0.35 ± 0.25	0.35 ± 0.25	0.35 ± 0.25
	**MS (1)**	-0.04 ± 0.38	-0.04 ± 0.38	-0.04 ± 0.38	-0.04 ± 0.38	0.24 ± 0.43	0.24 ± 0.43	0.24 ± 0.43	0.24 ± 0.43
**SF**	**HC (0)**	0.35 ± 0.15	0.35 ± 0.15	0.35 ± 0.15	0.35 ± 0.15	0.35 ± 0.13	0.35 ± 0.13	0.35 ± 0.13	0.35 ± 0.13
	**HC (1)**	0.20 ± 0.15	0.20 ± 0.15	0.20 ± 0.15	0.20 ± 0.15	0.12 ± 0.27	0.12 ± 0.27	0.12 ± 0.27	0.12 ± 0.27
	**MS (0)**	-0.35 ± 0.30	-0.35 ± 0.30	-0.35 ± 0.30	-0.35 ± 0.30	0.01 ± 0.47	0.01 ± 0.47	0.01 ± 0.47	0.01 ± 0.47
	**MS (1)**	-0.73 ± 0.29	-0.73 ± 0.29	-0.73 ± 0.29	-0.73 ± 0.29	-0.39 ± 0.41	-0.39 ± 0.41	-0.39 ± 0.41	-0.39 ± 0.41
**ST**	**HC (0)**	0.58 ± 0.23	0.58 ± 0.23	0.58 ± 0.23	0.58 ± 0.23	0.54 ± 0.05	0.54 ± 0.05	0.54 ± 0.05	0.54 ± 0.05
	**HC (1)**	0.55 ± 0.29	0.55 ± 0.29	0.55 ± 0.29	0.55 ± 0.29	0.62 ± 0.16	0.62 ± 0.16	0.62 ± 0.16	0.62 ± 0.16
	**MS (0)**	-0.34 ± 0.20	-0.34 ± 0.20	-0.34 ± 0.20	-0.34 ± 0.20	-0.11 ± 0.20	-0.11 ± 0.20	-0.11 ± 0.20	-0.11 ± 0.20
	**MS (1)**	-0.06 ± 0.21	-0.06 ± 0.21	-0.06 ± 0.21	-0.06 ± 0.21	0.04 ± 0.12	0.04 ± 0.12	0.04 ± 0.12	0.04 ± 0.12
**SYL**	**HC (0)**	0.27 ± 0.05	0.27 ± 0.05	0.27 ± 0.05	0.27 ± 0.05	0.26 ± 0.04	0.26 ± 0.04	0.26 ± 0.04	0.26 ± 0.04
	**HC (1)**	0.44 ± 0.46	0.44 ± 0.46	0.44 ± 0.46	0.44 ± 0.46	0.16 ± 0.28	0.16 ± 0.28	0.16 ± 0.28	0.16 ± 0.28
	**MS (0)**	0.25 ± 0.25	0.25 ± 0.25	0.25 ± 0.25	0.25 ± 0.25	0.15 ± 0.13	0.15 ± 0.13	0.15 ± 0.13	0.15 ± 0.13
	**MS (1)**	-0.12 ± 0.37	-0.12 ± 0.37	-0.12 ± 0.37	-0.12 ± 0.37	-0.08 ± 0.32	-0.08 ± 0.32	-0.08 ± 0.32	-0.08 ± 0.32
		**Left WM**				**Right WM**			
		**3D (A)**	**4D (A)**	**3D (B)**	**4D(B)**	**3D (A)**	**4D (A)**	**3D (B)**	**4D(B)**
**CALC**	**HC (0)**	0.52 ± 0.17	0.52 ± 0.17	0.52 ± 0.17	0.52 ± 0.17	0.45 ± 0.10	0.45 ± 0.10	0.45 ± 0.10	0.45 ± 0.10
	**HC (1)**	0.33 ± 0.33	0.33 ± 0.33	0.33 ± 0.33	0.33 ± 0.33	0.48 ± 0.23	0.48 ± 0.23	0.48 ± 0.23	0.48 ± 0.23
	**MS (0)**	0.39 ± 0.42	0.39 ± 0.42	0.39 ± 0.42	0.39 ± 0.42	0.15 ± 0.17	0.15 ± 0.17	0.15 ± 0.17	0.15 ± 0.17
	**MS (1)**	0.39 ± 0.39	0.39 ± 0.39	0.39 ± 0.39	0.39 ± 0.39	-0.02 ± 0.28	-0.02 ± 0.28	-0.02 ± 0.28	-0.02 ± 0.28
**CING**	**HC (0)**	0.07 ± 0.07	0.07 ± 0.07	0.07 ± 0.07	0.07 ± 0.07	0.00 ± 0.12	0.00 ± 0.12	0.00 ± 0.12	0.00 ± 0.12
	**HC (1)**	-0.34 ± 0.19	-0.34 ± 0.19	-0.34 ± 0.19	-0.34 ± 0.19	-0.68 ± 0.25	-0.68 ± 0.25	-0.68 ± 0.25	-0.68 ± 0.25
	**MS (0)**	0.25 ± 0.12	0.25 ± 0.12	0.25 ± 0.12	0.25 ± 0.12	0.06 ± 0.31	0.06 ± 0.31	0.06 ± 0.31	0.06 ± 0.31
	**MS (1)**	-0.32 ± 0.27	-0.32 ± 0.27	-0.32 ± 0.27	-0.32 ± 0.27	-0.66 ± 0.34	-0.66 ± 0.34	-0.66 ± 0.34	-0.66 ± 0.34
**CS**	**HC (0)**	0.10 ± 0.08	0.10 ± 0.08	0.10 ± 0.08	0.10 ± 0.08	0.21 ± 0.04	0.21 ± 0.04	0.21 ± 0.04	0.21 ± 0.04
	**HC (1)**	0.07 ± 0.25	0.07 ± 0.25	0.07 ± 0.25	0.07 ± 0.25	-0.14 ± 0.19	-0.14 ± 0.19	-0.14 ± 0.19	-0.14 ± 0.19
	**MS (0)**	0.14 ± 0.21	0.14 ± 0.21	0.14 ± 0.21	0.14 ± 0.21	0.20 ± 0.33	0.20 ± 0.33	0.20 ± 0.33	0.20 ± 0.33
	**MS (1)**	-0.22 ± 0.26	-0.22 ± 0.26	-0.22 ± 0.26	-0.22 ± 0.26	-0.10 ± 0.30	-0.10 ± 0.30	-0.10 ± 0.30	-0.10 ± 0.30
**PO**	**HC (0)**	0.34 ± 0.36	0.34 ± 0.36	0.34 ± 0.36	0.34 ± 0.36	0.12 ± 0.23	0.12 ± 0.23	0.12 ± 0.23	0.12 ± 0.23
	**HC (1)**	-0.05 ± 0.60	-0.05 ± 0.60	-0.05 ± 0.60	-0.05 ± 0.60	-0.24 ± 0.48	-0.24 ± 0.48	-0.24 ± 0.48	-0.24 ± 0.48
	**MS (0)**	0.03 ± 0.46	0.03 ± 0.46	0.03 ± 0.46	0.03 ± 0.46	0.23 ± 0.34	0.23 ± 0.34	0.23 ± 0.34	0.23 ± 0.34
	**MS (1)**	-0.29 ± 0.59	-0.29 ± 0.59	-0.29 ± 0.59	-0.29 ± 0.59	-0.11 ± 0.35	-0.11 ± 0.35	-0.11 ± 0.35	-0.11 ± 0.35
**SF**	**HC (0)**	0.23 ± 0.10	0.23 ± 0.10	0.23 ± 0.10	0.23 ± 0.10	0.23 ± 0.14	0.23 ± 0.14	0.23 ± 0.14	0.23 ± 0.14
	**HC (1)**	-0.05 ± 0.27	-0.05 ± 0.27	-0.05 ± 0.27	-0.05 ± 0.27	0.12 ± 0.23	0.12 ± 0.23	0.12 ± 0.23	0.12 ± 0.23
	**MS (0)**	0.11 ± 0.39	0.11 ± 0.39	0.11 ± 0.39	0.11 ± 0.39	0.38 ± 0.36	0.38 ± 0.36	0.38 ± 0.36	0.38 ± 0.36
	**MS (1)**	-0.01 ± 0.44	-0.01 ± 0.44	-0.01 ± 0.44	-0.01 ± 0.44	0.27 ± 0.49	0.27 ± 0.49	0.27 ± 0.49	0.27 ± 0.49
**ST**	**HC (0)**	0.37 ± 0.05	0.37 ± 0.05	0.37 ± 0.05	0.37 ± 0.05	0.27 ± 0.10	0.27 ± 0.10	0.27 ± 0.10	0.27 ± 0.10
	**HC (1)**	0.35 ± 0.14	0.35 ± 0.14	0.35 ± 0.14	0.35 ± 0.14	0.40 ± 0.57	0.40 ± 0.57	0.40 ± 0.57	0.40 ± 0.57
	**MS (0)**	0.17 ± 0.16	0.17 ± 0.16	0.17 ± 0.16	0.17 ± 0.16	0.40 ± 0.27	0.40 ± 0.27	0.40 ± 0.27	0.40 ± 0.27
	**MS (1)**	-0.17 ± 0.18	-0.17 ± 0.18	-0.17 ± 0.18	-0.17 ± 0.18	-0.07 ± 0.24	-0.07 ± 0.24	-0.07 ± 0.24	-0.07 ± 0.24
**SYL**	**HC (0)**	0.22 ± 0.09	0.22 ± 0.09	0.22 ± 0.09	0.22 ± 0.09	0.24 ± 0.15	0.24 ± 0.15	0.24 ± 0.15	0.24 ± 0.15
	**HC (1)**	-0.13 ± 0.29	-0.13 ± 0.29	-0.13 ± 0.29	-0.13 ± 0.29	-0.11 ± 0.41	-0.11 ± 0.41	-0.11 ± 0.41	-0.11 ± 0.41
	**MS (0)**	0.26 ± 0.24	0.26 ± 0.24	0.26 ± 0.24	0.26 ± 0.24	0.28 ± 0.10	0.28 ± 0.10	0.28 ± 0.10	0.28 ± 0.10
	**MS (1)**	0.14 ± 0.28	0.14 ± 0.28	0.14 ± 0.28	0.14 ± 0.28	0.19 ± 0.08	0.19 ± 0.08	0.19 ± 0.08	0.19 ± 0.08

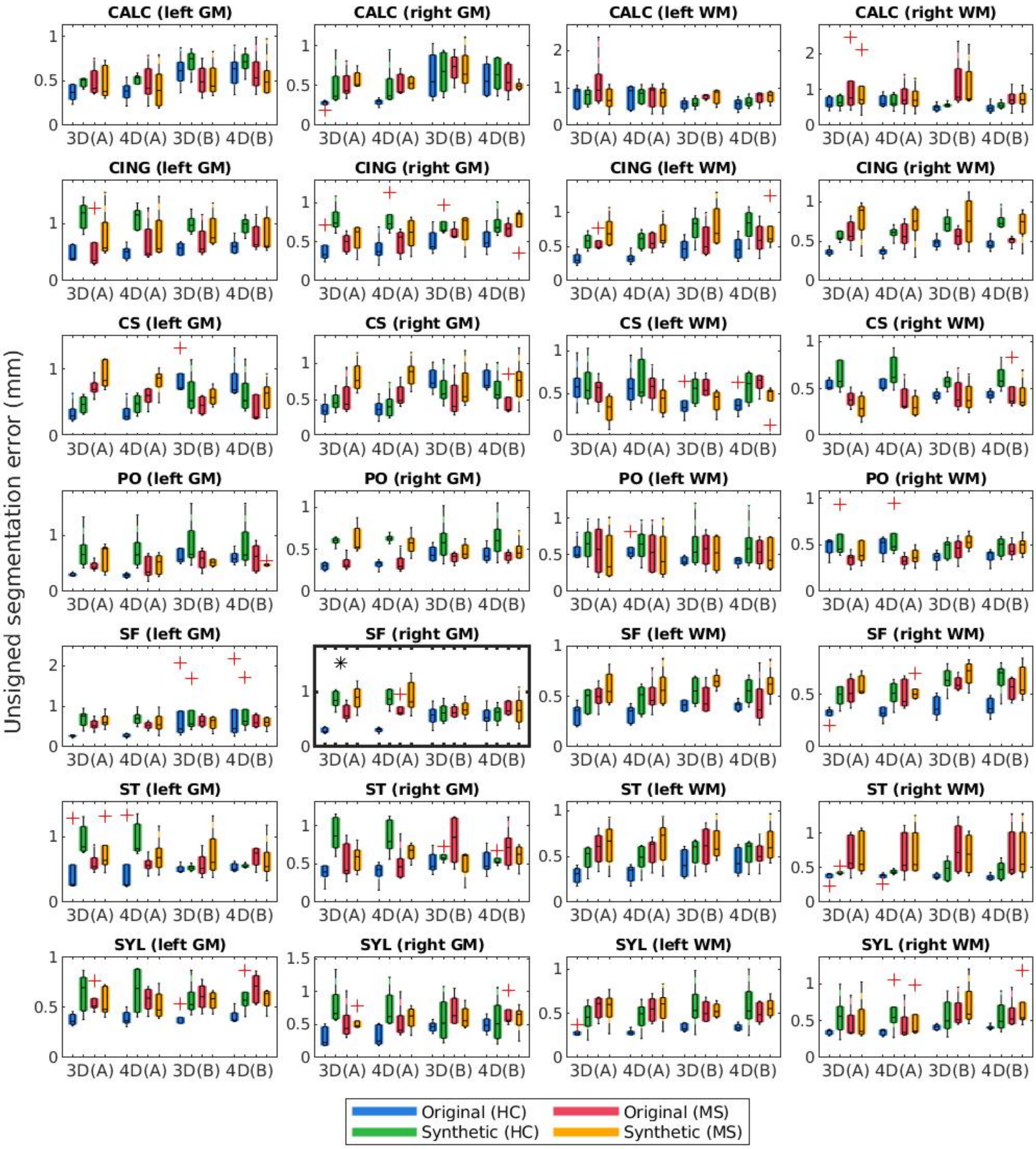

Figure S1. Mean unsigned segmentation errors of FreeSurfer cortical surface reconstructions for each set of landmarks across healthy and MS subjects. The horizontal axis for each subplot indicates the process type (cross-sectional or longitudinal) and the expert (A or B). Columns of subplots correspond to surface and hemisphere while the subplot row corresponds to the landmarks' anatomical placements. In each panel, mean segmentation errors from the original images of healthy subjects are shown in blue, synthetic images of healthy subjects in green, original images of MS subjects in red, and synthetic images from MS subjects in yellow.

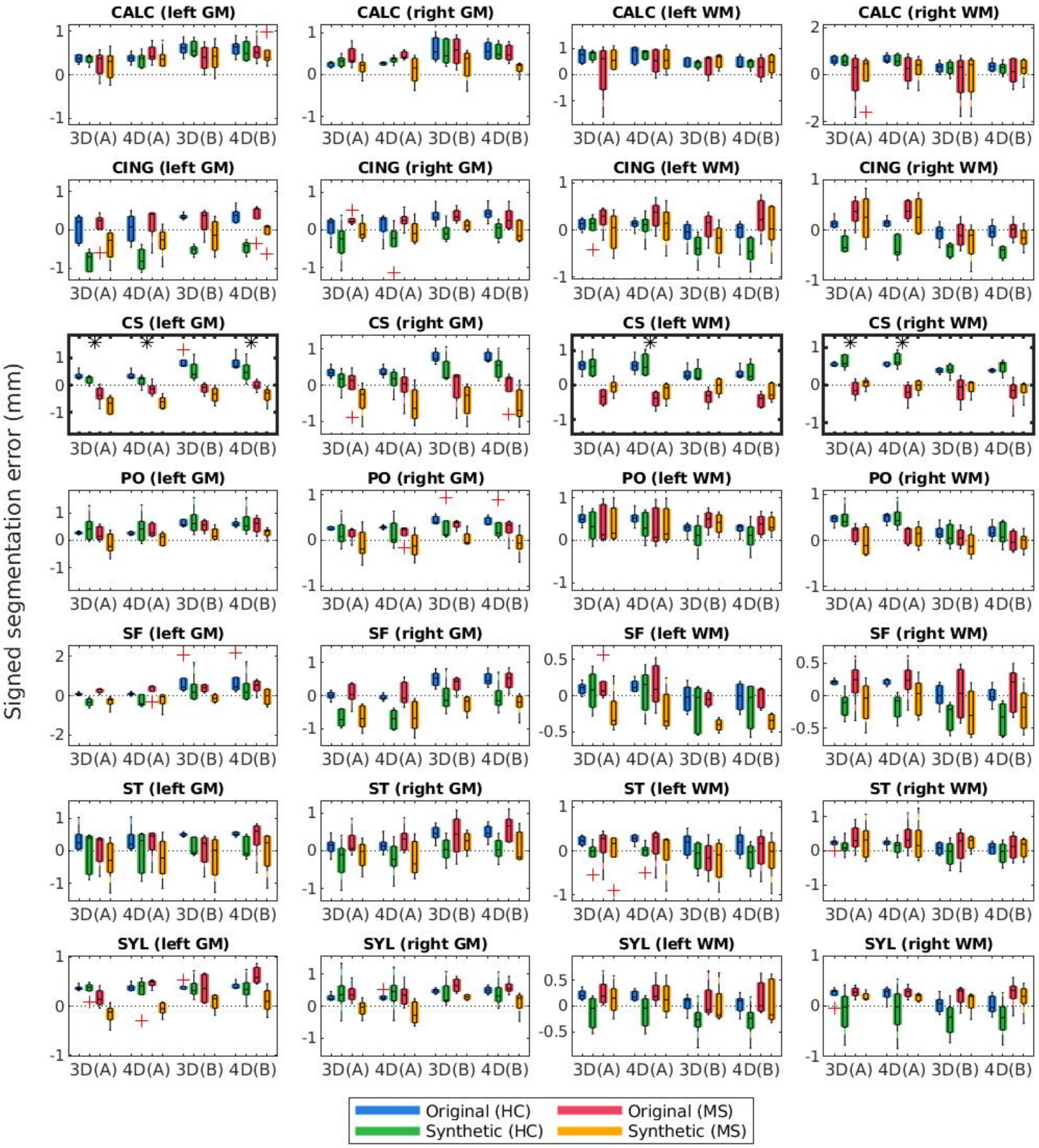

Figure S2. Mean signed segmentation errors of FreeSurfer cortical surface reconstructions for each set of landmarks across healthy and MS subjects. The horizontal axis for each subplot indicates the process type (cross-sectional or longitudinal) and the expert (A or B). Columns of subplots correspond to surface and hemisphere while the subplot row corresponds to the landmarks' anatomical placements. In each panel, mean segmentation errors from the original images of healthy subjects are shown in blue, synthetic images of healthy subjects in green, original images of MS subjects in red, and synthetic images from MS subjects in yellow.

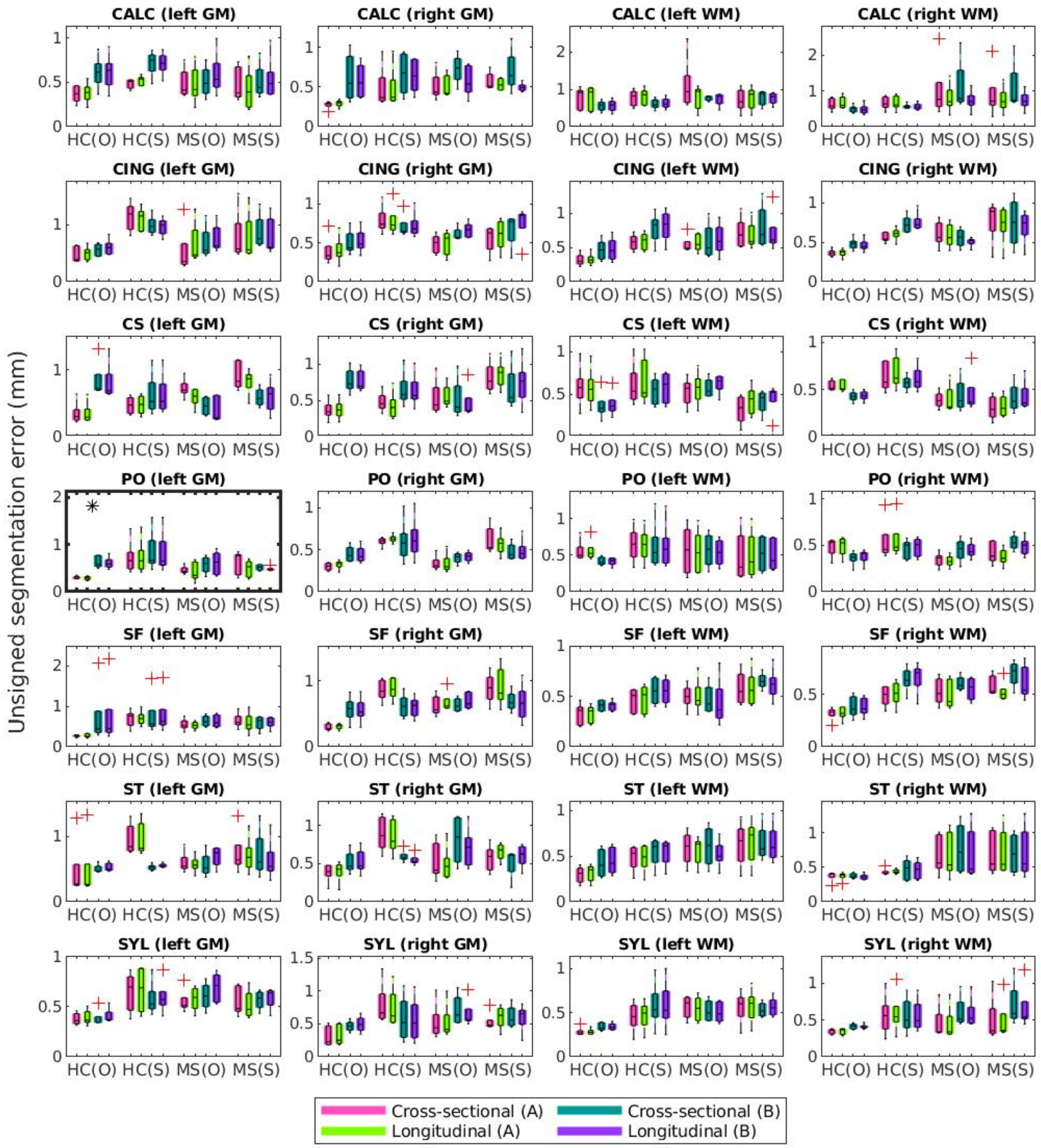

Figure S3. Mean unsigned segmentation errors of FreeSurfer cortical surface reconstructions for each set of landmarks across healthy and MS subjects. The horizontal axis for each subplot indicates the subject group (healthy control (HC) vs. MS) and the timepoint (0=original, 1=synthetic). Columns of subplots correspond to surface and hemisphere while the subplot row corresponds to the landmarks' anatomical placement. In each panel, mean segmentation errors from the cross-sectional method using landmarks placed by expert A are shown in pink, cross-sectional with expert B in teal, longitudinal with expert A in green, and longitudinal with expert B in purple.

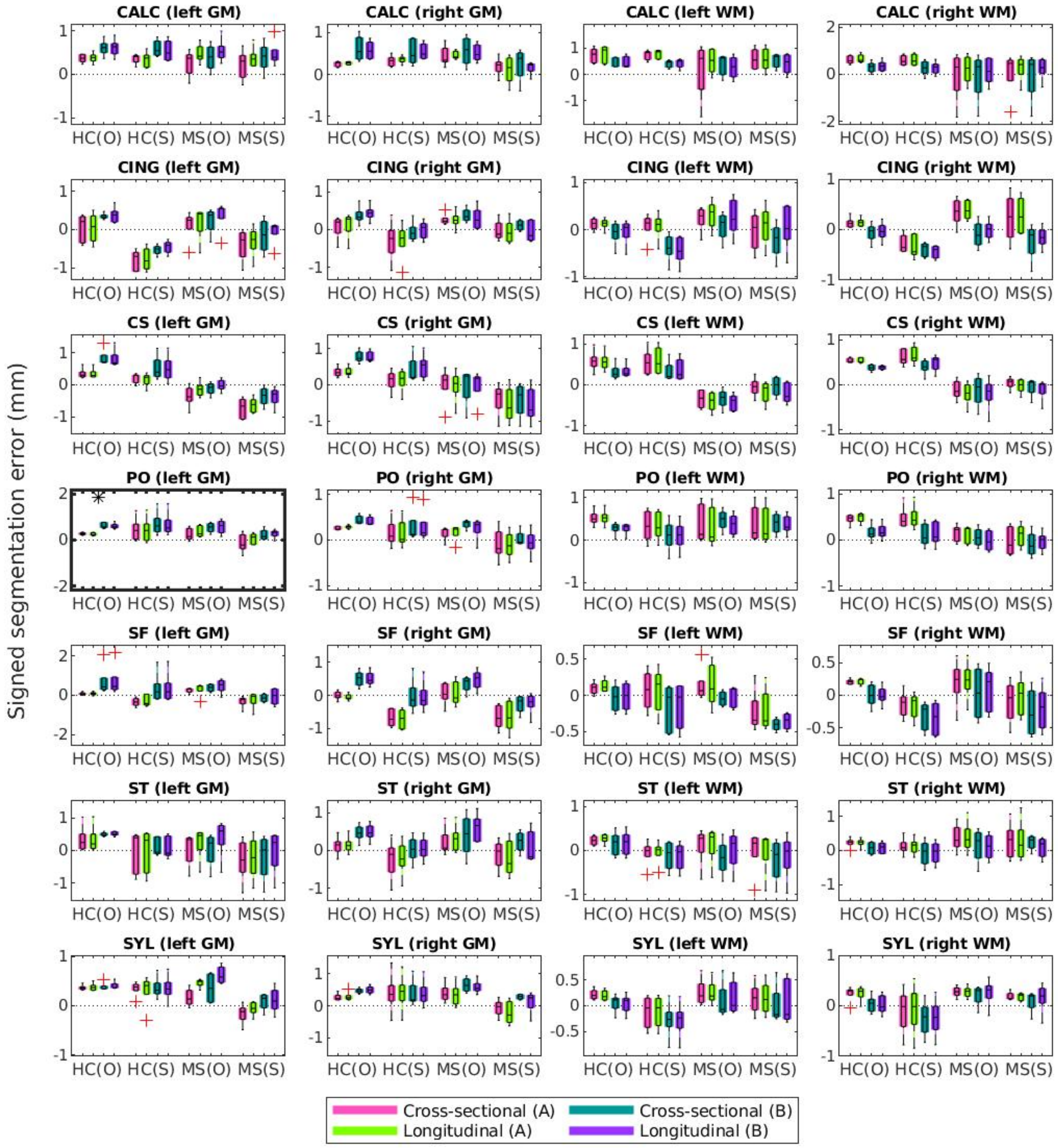

Figure S4. Mean signed segmentation errors of FreeSurfer cortical surface reconstructions for each set of landmarks across healthy and MS subjects. The horizontal axis for each subplot indicates the subject group (healthy control (HC) vs. MS) and the timepoint (0=original, 1=synthetic). Columns of subplots correspond to surface and hemisphere while the subplot row corresponds to the landmarks' anatomical placement. In each panel, mean segmentation errors from the cross-sectional method using landmarks placed by expert A are shown in pink, cross-sectional with expert B in teal, longitudinal with expert A in green, and longitudinal with expert B in purple.
